# HPV16 E7 Protein and hTERT Proteins Defective for Telomere Maintenance Cooperate to Immortalize Human Keratinocytes

**DOI:** 10.1371/journal.ppat.1003284

**Published:** 2013-04-04

**Authors:** Jonathan Miller, Aleksandra Dakic, Renxiang Chen, Nancy Palechor-Ceron, Yuhai Dai, Bhaskar Kallakury, Richard Schlegel, Xuefeng Liu

**Affiliations:** Department of Pathology, Georgetown University Medical Center, Washington, D.C., United States of America; University of Virginia, United States of America

## Abstract

Previous studies have shown that wild-type human telomerase reverse transcriptase (hTERT) protein can functionally replace the human papillomavirus type 16 (HPV-16) E6 protein, which cooperates with the viral E7 protein in the immortalization of primary keratinocytes. In the current study, we made the surprising finding that catalytically inactive hTERT (hTERT-D868A), elongation-defective hTERT (hTERT-HA), and telomere recruitment-defective hTERT (hTERT N+T) also cooperate with E7 in mediating bypass of the senescence blockade and effecting cell immortalization. This suggests that hTERT has activities independent of its telomere maintenance functions that mediate transit across this restriction point. Since hTERT has been shown to have a role in gene activation, we performed microarray studies and discovered that E6, hTERT and mutant hTERT proteins altered the expression of highly overlapping sets of cellular genes. Most important, the E6 and hTERT proteins induced mRNA and protein levels of Bmi1, the core subunit of the Polycomb Group (PcG) complex 1. We show further that Bmi1 substitutes for E6 or hTERT in cell immortalization. Finally, tissue array studies demonstrated that expression of Bmi1 increased with the severity of cervical dysplasia, suggesting a potential role in the progression of cervical cancer. Together, these data demonstrate that hTERT has extra-telomeric activities that facilitate cell immortalization and that its induction of Bmi1 is one potential mechanism for mediating this activity.

## Author Summary

The human papillomaviruses (HPVs) are critical elements in the etiology of cervical cancer, as well as several other human cancers. The E6 protein, in combination with the E7 protein of these viruses, immortalizes epithelial cells and increases the expression of the hTERT protein. In the current study we show that the enzymatic activity of hTERT is not required for cooperating in cell immortalization. We further demonstrate that hTERT proteins increase the expression of the Bmi1 protein, which is also capable of cooperating in cell immortalization. We anticipate that these findings will stimulate new studies of telomerase in HPV biology, cancer etiology, and stem cell reprogramming.

## Introduction

Cell immortality is a hallmark of cancer cells [1] and the high-risk oncogenic HPVs encode two major transforming genes, E6 and E7, which are required for the immortalization of human primary genital keratinocytes [2], [3]. These two oncogenes are uniformly retained and expressed in cervical cancers and their continued expression is required for the cells to retain the tumorigenic phenotype [4], [5], [6], [7], [8]. The E6 and E7 proteins were initially identified as targeting the p53 and Rb tumor suppressor pathways in host cells, thereby disrupting cell cycle controls [5], [6], [7], [8]. E7 stimulates the cell cycle via its ability to bind and inactivate the cellular Rb protein while E6 binds to p53, leading to its degradation via the proteosomal pathway [5], [6], [7], [8].

In addition to p53 degradation, E6 induces telomerase activity in epithelial cells [6], [9], [10]. Telomerase is a specialized reverse transcriptase that synthesizes the telomeric repeat DNA sequences at the ends of chromosomes [11]. The absence of telomerase activity in most normal human cells results in the progressive shortening of telomeres with each cell division [12], [13], ultimately leading to chromosomal instability and cellular replicative senescence [12], [14]. For this reason, telomere shortening is thought to represent the “mitotic clock” that determines cellular lifespan. In contrast to most human somatic cells, approximately 90% of immortalized and cancer cells express telomerase activity and consequently maintain minimal, stable telomeres and indefinite proliferative potential [15]. Therefore, telomerase activation is considered a critical event in the process of cell immortalization. Recent studies indicate that telomerase may assist in bypassing two separate events which block the continuous replication of primary human cells: mortality stage 1 (M1, replicative senescence) followed by mortality stage 2 (M2, crisis) [16]. In some cells, especially those with decreased function of the p16/Rb pathway, telomerase activity is sufficient to bypass both M1 and M2 blockades and to stabilize and elongate telomeres [17], [18], [19], [20].

Studies have demonstrated that activation of telomerase by E6 is critical for cell immortalization by HPV [17], [21]. E6 executes this increase in telomerase activity by multiple mechanisms [8], [22], [23], [24], [25], [26]. While increased hTERT is required for viral-mediated cell immortalization [8], [17], [21], our previous studies demonstrated that telomeres erode in HPV-expressing keratinocytes similar to normal keratinocytes [10], suggesting that the role of hTERT overexpression in cell immortalization might involve functions additional to those in telomere elongation.

Evidence is accumulating that hTERT has important non-canonical functions. For example, mTERT has been ascribed roles in altering apoptotic responses [27], [28], tumor formation in mice [29], [30], stem cell migration and renewal [30] and chromatin remodeling [31]. The Artandi laboratory has shown that mTERT can not only augment breast cancer development in mice, but also can regulate the transcription of genes responsive to the Wnt/β-catenin pathway [30]. Smith *et al* demonstrated that in human mammary epithelial cells (hMECs) telomerase modulates expression of growth-controlling genes, including epidermal growth factor receptor (EGFR) [32]. Vascular endothelial growth factor (VEGF) and fibroblast growth factor (FGF) also appear to be induced by hTERT in fibroblasts, along with many other targets [33], [34]. Interestingly, the majority of these data have been recapitulated with an hTERT mutant that is catalytically-inactive, suggesting that these non-canonical roles of hTERT are independent of the reverse transcriptase function.

Although hTERT has been shown to be a key player in cellular immortalization, in many cases it does not immortalize alone [17]. Interestingly, the Bmi1 protein has been shown to cooperate with hTERT in immortalization and to induce hTERT mRNA [35], [36]. Bmi1 is the central protein in polycomb repressive complex 1 (PRC1). The Polycomb group (PcG) complex of proteins act through remodeling chromatin to silence hundreds of genes and have been implicated in controlling cell fate, development, and cancer [37], [38].

In the current study, we used quantitative assays to measure telomerase activity and telomere length following transduction of foreskin keratinocytes by E6/E7, hTERT/E7 and mutant hTERT/E7. These activities were correlated with the ability of the various hTERT mutant proteins to immortalize cells. Our studies indicate that a telomerase-independent activity of hTERT collaborates with E7 in the immortalization of primary human cells. To elucidate the underlying mechanism, whole genome expression profiling was performed in keratinocytes expressing E6, hTERT, or a catalytically inactive hTERT mutant (hTERT-D868A). Increased expression of Bmi1 mRNA was observed in this screening and follow-up experiments indicate that Bmi1 appears to be a functional component of hTERT- or E6-mediated cell immortalization and that its expression further increases during cancer progression.

## Results

### HPV-immortalized and HPV cancer cells have equivalent high telomerase activity but short telomeres

**Figure 1:**
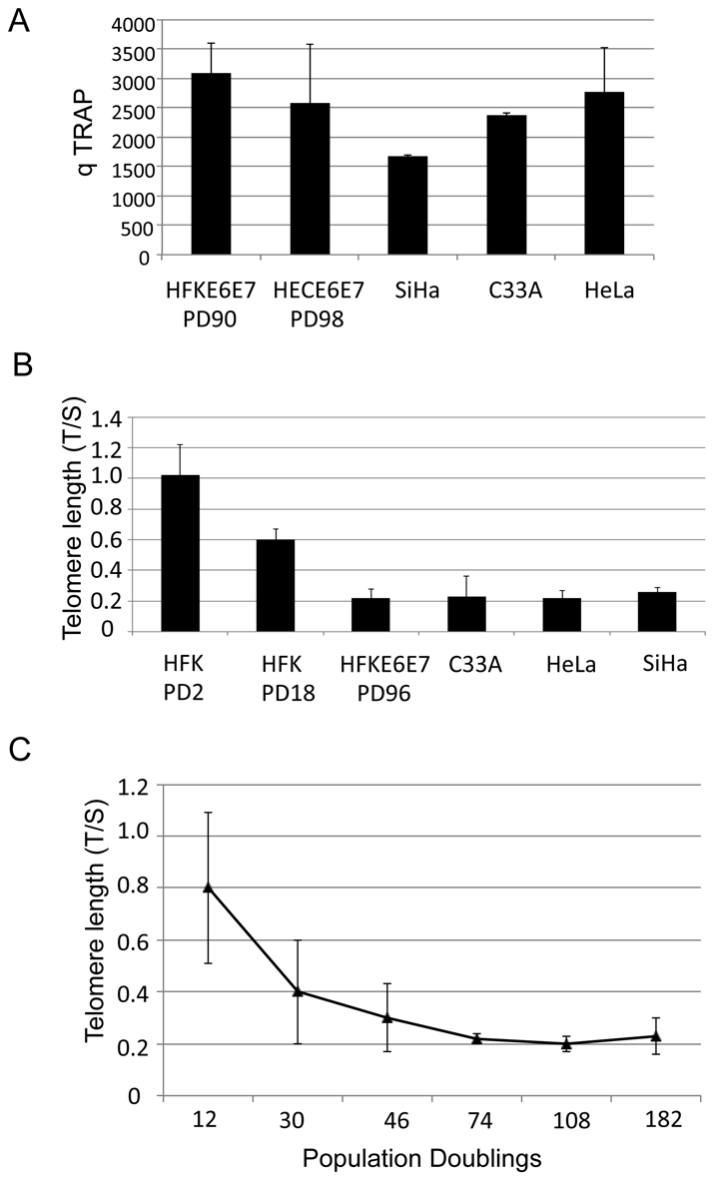
Telomerase activity does not correlate with telomere length during immortalization of human genital keratinocytes by the HPV E6 and E7 oncoproteins. Primary human foreskin keratinocytes (HFKs) and human ectocervical keratinocytes (HECs) were transduced with pLXSN-based retroviruses containing HPV E6, E7, E6/E7, or empty vector and selected as previously described. Cultures were passed continuously in vitro as described in the text and the number of cell doublings calculated and plotted versus time in culture. Cultures that did not proliferate and expand in 20 days were considered senescent and were terminated. This experiment was repeated more than five times with similar results. (A) Immortalized cells exhibit similar levels of telomerase activity as in cervical cancer cells. Quantitative TRAP assays as described in the Materials and Methods were used to measure telomerase activity in E6/E7 immortalized HFKs, E6/E7 immortalized HECs, and SiHa (HPV-16 positive), HeLa (HPV-18 positive), and C33A (HPV negative) cervical cancer cell lines. (B) Telomere length stabilizes in E6/E7 immortalized cells and cervical cancer cell lines. Cellular DNAs were isolated from HFKs at indicated passages and cervical cancer cells, and relative telomere length (T/S ratio = telomere/single copy gene) was measured using real-time PCR, as described in the Material and Methods. Immortalized cells and cancer cells have relatively shorter telomeres. (C) Telomere length shortens over cell passages during immortalization. Cellular DNAs were isolated and subjected to real-time PCR-based telomere length measurement.

Immortalized cells generally do not display the phenotypic properties of cancer cells (e.g. growth in soft agar or tumor formation in nude mice) without further gain of genetic changes or transduction of additional genes [5], [8], [39]. To determine if changes in telomerase activity might contribute to the differences between immortalized and tumorigenic cervical cells, we compared the levels of telomerase activity for cells immortalized by the HPV-16 E6/E7 genes to those found in 3 cervical cancer cell lines (SiHa, HPV16 positive; HeLa, HPV-18 positive; C33A, HPV negative). The E6/E7 immortalized cell lines (HFK E6/E7 at population doubling (PD) 90 and HEC E6/E7 at PD 98) exhibited similar levels of telomerase activity as the three cervical cancer lines, indicating that further increases in telomerase are not required for progression to malignancy (**Figure 1A**).

While an early hypothesis for HPV-mediated cell immortalization suggested that telomerase induction by E6 maintained telomere length [8], [9], [17], our previous studies showed that E6/E7 expressing cells continued to shorten telomeres even in the presence of induced telomerase activity [10]. To quantify these changes in telomere length, we used a PCR-based assay to screen E6/E7 immortalized cells and cervical cancer cell lines. At PD 2, HFKs had long average telomere lengths with a T/S ratio of 1.0 (**Figure 1B**). Since the approximate length of telomeres in early passage HFKs is 10 kb, the T/S ratio can be converted into telomere length (where 1.0 T/S ratio equals 10 kb telomere length). At PD 18, HFKs had a T/S ratio of 0.6 or 6 kb size. Immortalized, PD 96 HFK E6/E7 cells, which have bypassed crisis, had amongst the shortest telomeres (**Figure 1B**; T/S ratio of 0.2, or 2 kb length). These short telomeres were also seen in all three cervical cancer cell lines, including the HPV-negative cancer cell line, C33A. Our data suggest that E6/E7-immortalized cells continue to degrade their telomeres until they reach a length of 2 kb, at which point they become stabilized and equivalent in length to telomeres in cervical cancer cell lines.

The kinetics of passage-dependent shortening or degradation of telomeres during cell immortalization by E6/E7 were also studied (**Figure 1C**). By PD 74, HFK cells expressing E6/E7 achieved their shortest telomere length, after which telomere length became stable.

### A telomere association-defective hTERT protein, hTERT-HA, cooperates with E7 to immortalize human keratinocytes

An hTERT protein that was epitope-tagged at its C-terminus (hTERT-HA) retained telomerase activity, but alone was unable to elongate telomeres or immortalize HFFs or SV40 transformed epithelial cells [40], [41], [42]. To test the functions of wild-type hTERT and hTERT-HA in human keratinocytes, HFKs were co-transduced with vectors expressing E7 and the hTERT proteins. As expected, wild-type hTERT cooperated with E7 to immortalize HFKs, while hTERT or E7 alone were unable to immortalize HFKs. Surprisingly, hTERT-HA also immortalized HFKs in collaboration with E7 [26], indicating that telomere maintenance is not critical for hTERT/E7 immortalization. The functionality of E7 was verified by demonstrating that the Rb protein level was significantly decreased in all E7 expressing cells (**Figure S1**).

**Figure 2:**
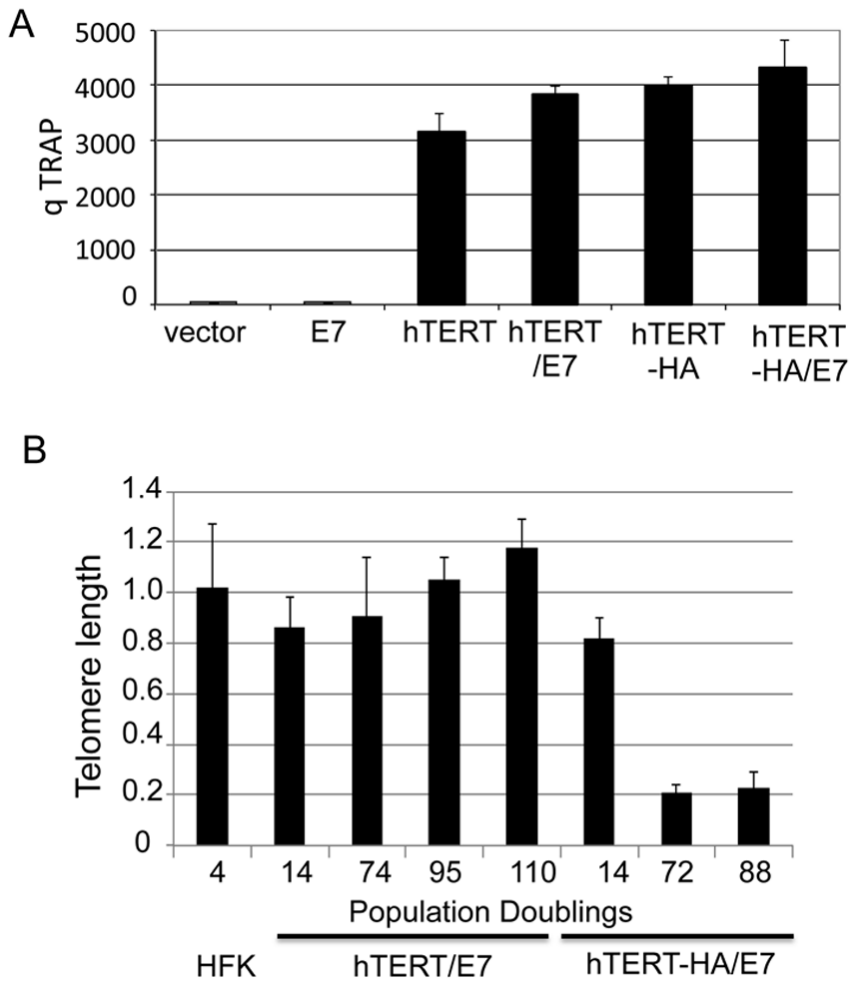
A telomere elongation-defective hTERT mutant cooperates with HPV E7 for immortalizing human keratinocytes (HFKs). Primary HFKs were transduced with pBABE-puro-based retroviruses containing hTERT or hTERT-HA and pLXSN-based retroviruses with HPV E7 or empty vector and selected with puromycin and G418 as previously described. Cultures were passed continuously in vitro, and growth curves were plotted. Cultures that did not proliferate and expand in 20 days were considered senescent and were terminated. This experiment was repeated more than three times with similar results. (A) Telomerase activity. Quantitative TRAP assays were done as described in the Materials and Methods. Similar levels of telomerase activity were observed among cells expressing an hTERT construct. (B) Telomere length. Telomeres lengthened in hTERT/E7 cells, but shortened in hTERT-HA/E7 cells. Both wild-type hTERT and hTERT-HA immortalize HFKs in combination with HPV E7.

To verify that the hTERT-HA mutant generated telomerase activity in HFKs, Telomeric Repeat Amplification Protocol (TRAP) assays were performed. HFKs transduced with hTERT or hTERT-HA alone, or in combination with E7, exhibited similar levels of telomerase activity (**Figure 2A**). HFKs with E7 alone did not exhibit significant telomerase activity. Consistent with our earlier results, **Figure 2B** illustrates that telomeres lengthened during immortalization of the hTERT/E7 cells, but shortened in the hTERT-HA/E7 cells (**Figure 2B**).

### hTERT proteins mutated in the catalytic and telomere recruitment domains retain immortalizing activity

**Figure 3:**
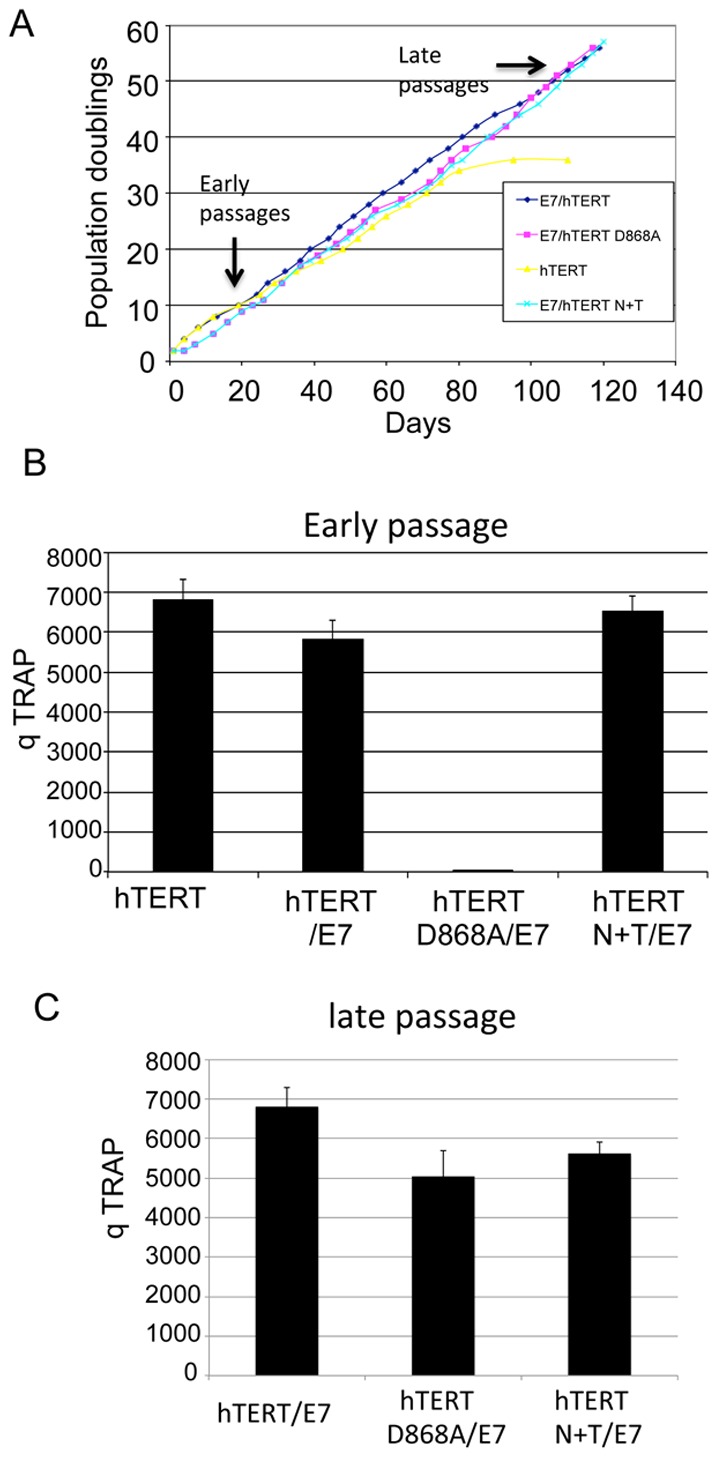
Catalytic-defective hTERT mutants cooperate with HPV E7 to immortalize HFKs. (A) Growth curves. Primary HFKs were transduced with the indicated pBABE-puro based retroviruses containing wild-type hTERT, hTERT N+T, or hTERT-D868A and pLXSN-based retroviruses containing E7 or empty vector and then doubly selected with puromycin and G418 as previously described. Cultures were passed continuously in vitro and growth curves were plotted with population doubling over time in culture. Cultures that did not proliferate and expand in 20 days were considered senescent and were terminated. This experiment was repeated more than three times with similar results. Wild-type hTERT, hTERT-D868A, and hTERT N+T are all able to immortalize HFKs in combination with E7. (B) Telomerase activity in early passage of the transduced cells. CHAP lysates were harvested from early (p5) and telomerase activity was measured by quantitative real-time TRAP. (C) Telomerase activity in late passage of the transduced cells. Telomerase activity in late passage of cells was measured by quantitative real- time TRAP.

The preceding experiments indicate that cell immortalization is independent of telomere lengthening and raise the possibility that other telomere-related functions of hTERT were involved in this process. To evaluate this possibility, we therefore examined the immortalizing activity of additional hTERT mutants that were known to be catalytically inactive (hTERT-D868A) [43] or had impaired recruitment to telomeres (hTERT N+T) [42], [44]. Similar to hTERT-HA, both the hTERT-D868A and hTERT N+T mutants were able to immortalize keratinocytes in conjunction with E7 (**Figure 3A**). Cells immortalized by the hTERT N+T mutant exhibited similar levels of telomerase activity as cells immortalized by E6/E7, so decreased telomerase activity could be ruled out as a potential mechanism (**Figure 3B**). The results with hTERT-D868A were even more significant. Cells immortalized by this defective mutant were as efficient at cell immortalization as wild-type hTERT (**Figure 3A**), despite the complete lack of telomerase activity in early passage keratinocytes (**Figure 3B**). Immunofluorescence studies demonstrated that hTERT-D868A exhibited a similar expression level and localization as wild-type hTERT (**Figure S2**). Thus, the catalytic activity of hTERT and its ability to elongate telomeres is dispensable for the immortalization of keratinocytes with E7.

**Figure 4:**
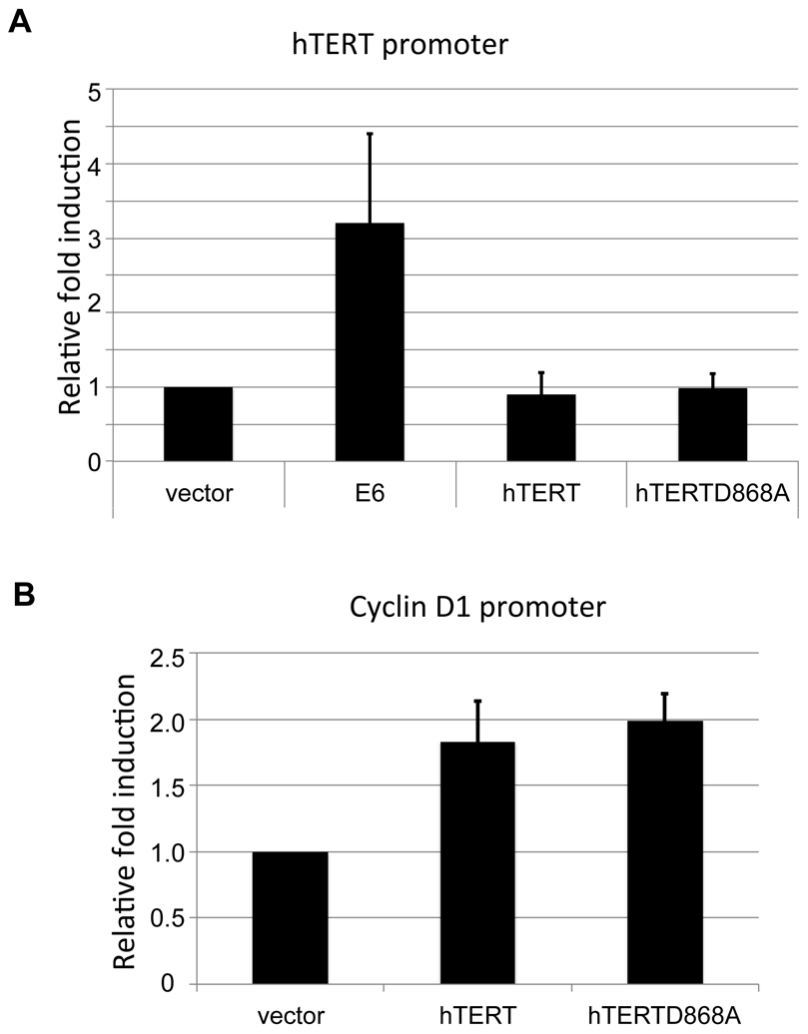
hTERT wt and hTERT-D868A do not activate the hTERT promoter. Keratinocytes were transfected with either wt hTERT core promoter or the cyclin D1 promoter and either HPV16E6, hTERT wt, or hTERT-D8686A. The pRL-CMV *R. reniformis* reporter plasmid was also transfected into the cells to standardize for transfection efficiency. Luciferase activity was measured 24 hours after transfection using the Dual luciferase reporter assay system (Promega). Relative fold activation reflects the normalized luciferase activity induced by E6 and hTERT compared to the normalized activity of vector control. The value of pGL3B-hTERT activity with empty was set to 1. Error bars show the standard deviation for at least three independent experiments. Neither hTERT wt nor hTERT-D868A induce hTERT core promoter (A), while they are able to activate cyclin D1 promoter (B). HPV16 E6 was as a positive control for induction of hTERT promoter.

Another unexpected finding was that cells immortalized by the telomerase-defective hTERT-D868A mutant exhibited high telomerase activity at late passages (**Figure 3C**) in contrast to the lack of telomerase at early passages (**Figure 3B**). This led us to ask whether hTERT proteins, including hTERT-D868A activate the endogenous hTERT promoter. To test this, we transfected wild type or mutant hTERT proteins along with an hTERT core promoter construct into HFK. The data from luciferase reporter assays demonstrated that neither wild type hTERT nor mutant hTERT-D868A activated the hTERT promoter (**Figure 4A**). This is consistent with the lack of endogenous telomerase activity in early-passage cells transduced with the hTERT-D868A mutant (**Figure 3B**). HPV E6 was used as positive control in this experiment (**Figure 4A**). We also confirmed that both wild type and mutant hTERT proteins were biologically active in this assay and able to activate the cyclin D promoter (**Figure 4B**) as described previously [30]. Together, these findings suggest that inactive hTERT proteins (in collaboration with E7) can mediate transit through crisis (the M2 phase of cell), but that continued cell proliferation correlates with increased endogenous hTERT expression, identical to what is observed in cells immortalized by the E6 protein. The implications of these findings are considered in the Discussion.

### Wild-type and mutant hTERT alter gene expression profiles similar to HPV E6

**Figure 5:**
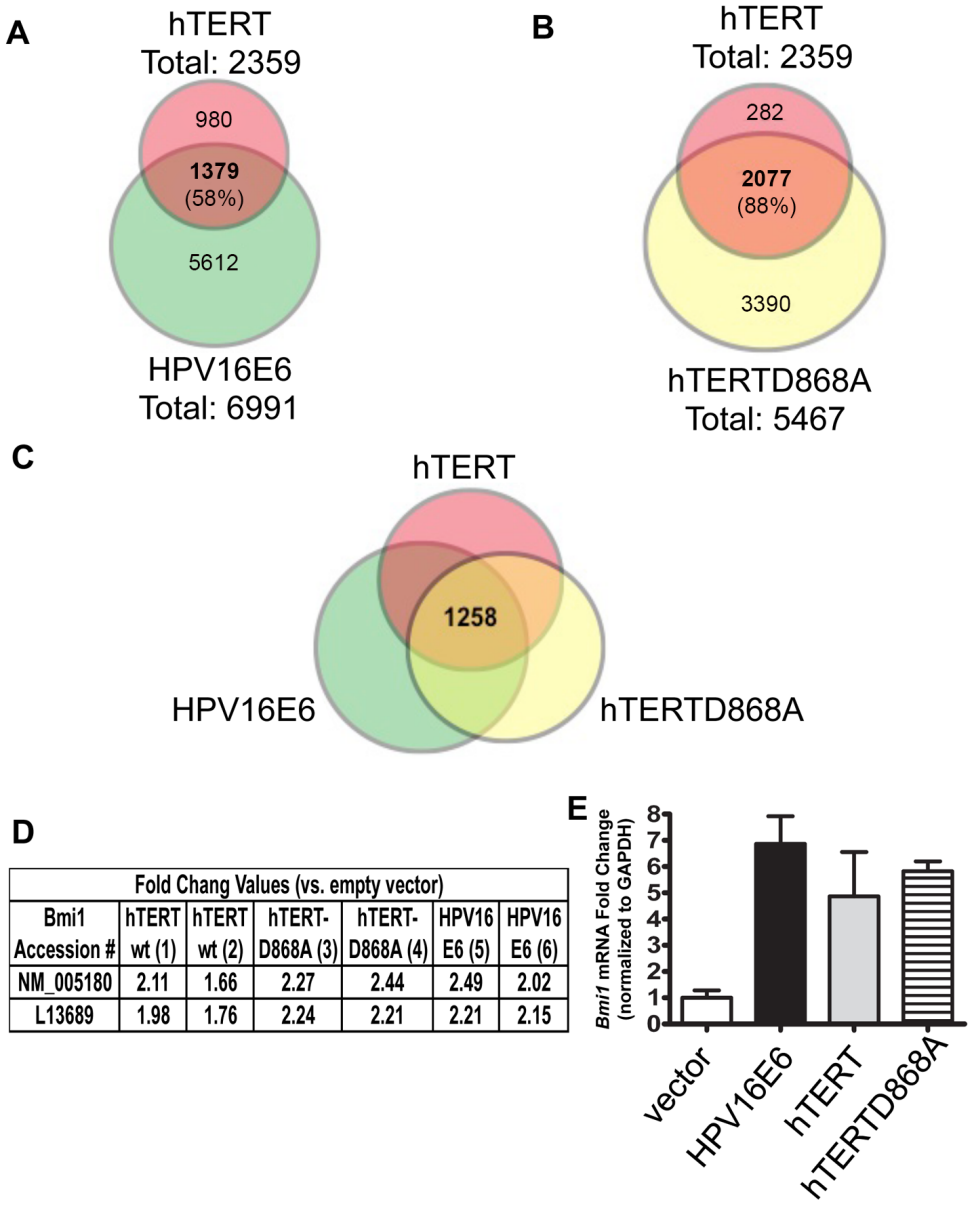
E6, hTERTwt, and hTERT-D868A alter the expression of overlapping gene sets, including chromatin remodeling genes such as *Bmi1*. Primary HFKs were stably transduced with either E6, hTERTwt, hTERT-D868A or puro babe control vector. Samples were submitted for whole genome expression array analysis (**Figure S3**). Expression profile changes shown by hTERTwt are compared to expression profile changes in E6 (**A**) and hTERT-D868A mutant (**B**). As expected, a significant amount of the expression changes seen in E6 (6991 total changes) were also altered by hTERT wt (1379, representing 20% of the E6 changes). Conversely, more than half of the hTERT changes (58%, 1379 of 2359 changes) were also seen by E6. Interestingly, of the 2359 genes altered by hTERTwt, 2077 of them (88%) were also altered by the hTERT^ci^ mutant (fold change >1.33 and *p* value <0.01). (**C**) 1258 changes were shared by E6, hTERTwt, and hTERT-D868A, including Bmi1. (**D**) Numerical fold change values are shown for all arrays as they correspond to two probes, NM_005180 and L13689. These accession numbers represent Bmi1 mRNA sequences that are 99% identical. Quantitative RT-PCR was performed on the E6 and hTERTwt or hTERT-D868A (**E**) with gene-specific primers for Bmi1 to validate the array results, normalized to GAPDH. n = 3. Bars represent mean ± SD.

Since previous studies have defined extra-telomeric functions of hTERT, we attempted to identify a potential telomere-independent mechanism to explain the ability of the inactive hTERT to immortalize cells, with a specific focus on cellular gene expression. Given the conflicting reports of hTERT on gene expression in various model systems [32], [33], [34], [45], [46], [47], [48], it was important that we examined hTERT effects in primary keratinocytes. We therefore stably expressed E6, wild-type hTERT (hTERTwt), or hTERT-D868A in primary HFKs and conducted array-based whole genome expression analysis (**Figure S3**, and **Dataset S1**). Because E6 is a known activator of the hTERT protein [8], expression changes shared by hTERT and E6 could represent hTERT-dependent E6 targets. As expected, significant changes in mRNA expression in E6 cells were also seen in cells with wt hTERT (1379 of 6991, or 20% of E6 changes with fold change >1.33 and *p*-value <0.01) (**Figure 5A**). More than half of the wt hTERT changes (58%, 1379/2359) were also seen in E6 cells, suggesting that changes seen in hTERT-expressing cells are also altered by E6-expressing cells, possibly through an hTERT-dependent pathway.

To pursue whether the mRNA expression changes seen in wt hTERT cells were dependent on changes in telomere biology, we also expressed the catalytically inactive mutant hTERT-D868A in primary HFKs. A total of 2359 mRNA probe sets were altered in wt hTERT HFKs compared to 5467 changes in hTERT-D868A HFKs (**Figure 5B**). Interestingly, 2077 of the 2359 (88%) of the RNA probes altered by wt hTERT were also altered by hTERT-D868A. Thus, the gene expression alterations seen following hTERT expression are largely independent of reverse transcriptase function.

### Bmi1 increases with expression of wild type hTERT

**Figure 6:**
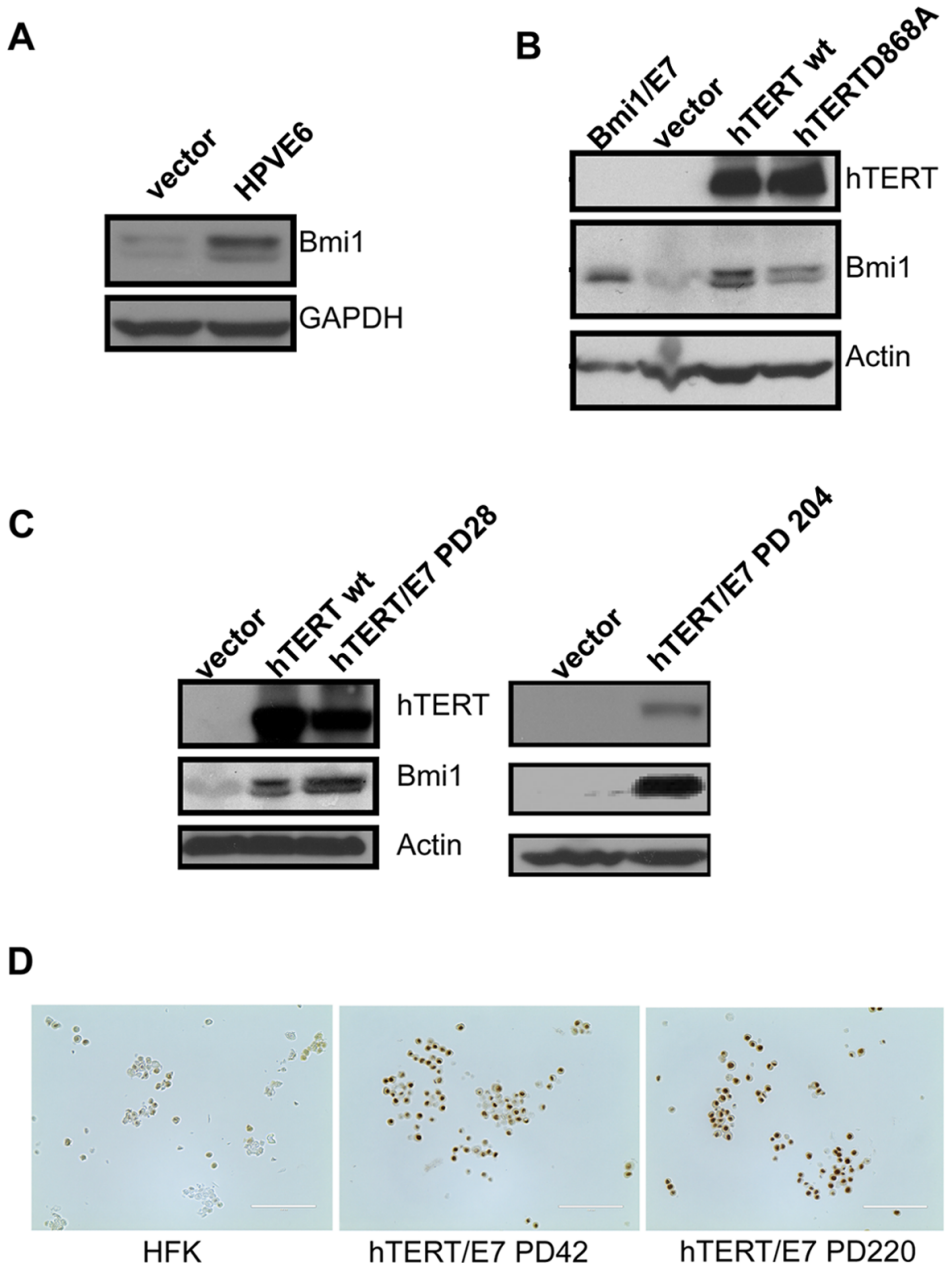
Bmi1 protein increases in cell lines. Bmi1 protein levels were quantified by Western blot for (**A**) empty vector and E6 expressing cells and (**B**) hTERTwt or hTERT-D868A expressing cells. Bmi1+E7 lysate served as a positive control for Bmi1 protein. Lysates were separated by 4–20% gradient SDS-PAGE. Antibodies were used to detect hTERT (1∶1000, Origene) and Bmi1 (1∶200, F6, Millipore), normalized to GAPDH (1∶2000, FL-335, Santa Cruz) or ACTIN (1∶5000, Sigma). (**C**) Bmi1 protein levels were quantified by Western blot. Long-term serial passaging maintains increased levels of Bmi1 protein, as shown by the P102 hTERT+E7 lysate. Lysates were separated by 4–20% gradient SDS-PAGE. Antibodies were used to detect hTERT (1∶1000, Origene), Bmi1 (1∶200, F6, Millipore) and actin (1∶5000, Sigma). (**D**) Cell pellets were formalin fixed and paraffin embedded. Bmi1 protein levels were assayed by immunocytochemistry (1∶200, F6, Millipore). The images were captured with the Evos LX microscope. Scale bar = 100 µm.

1258 changes in mRNA expression were shared by wt hTERT, hTERT-D868A, and E6 (**Figure 5C** **, Dataset S1**). Thus, additional considerations were required to focus our study (**Figure 5C**). The wt hTERT/hTERT-D868A overlapping gene set was submitted for analysis using Database for Annotation, Visualization and Integrated Discovery (DAVID) [49]. Hierarchical clustering of the 2077 catalytically-independent changes was used to identify 408 gene clusters that were visualized as a heatmap [50] (**Figure S4**). Based on enrichment scores, genes associated with “Chromosome Organization”, “Chromatin Organization”, and “Chromatin Modification” were grouped with the SP-PIR Keyword “Chromatin Regulator” (**Dataset S2**). Of particular interest in the enriched chromatin regulation cluster was the gene *Bmi1*, which exhibited significant increases in two overlapping probe sets (**Figure 5D**, probe set IDs L13689 and NM_005180). We verified by RT-PCR that E6, wt hTERT and hTERT-D868A expression led to 5–7 fold increases in Bmi1 transcript levels (**Figure 5E**). More important, these three genes also increased Bmi1 protein expression (**Figure 6A, B**).

To further validate the RT-independent ability of hTERT to induce Bmi1, we analyzed two additional mutated hTERT constructs that lacked telomerase activity. Both of these telomerase mutants increased Bmi1 transcript levels, similar to hTERT-D868A mutant (**Figure S5**), further substantiating the telomerase-independent activity involved in Bmi1 induction.

### Bmi1 remains increased in immortalized keratinocytes

Since Bmi1 expression is increased acutely by hTERT, we investigated whether Bmi1 levels remained increased in late-passage HFKs immortalized by hTERT/E7. We doubly transduced HFKs with hTERT and E7 and propagated the cells beyond the time when they would normally enter crisis. Bmi1 protein was shown to be increased in early passage (28 population doublings, PD 28) hTERT/E7 immortalized HFKs and remained high after serial passaging (PD 204) (**Figure 6C**). We also confirmed increased Bmi1 protein expression in hTERT/E7 immortalized HFKs by immunohistochemistry (**Figure 6D**). Compared to control HFKs, Bmi1 was significantly increased in the nuclei of early passage (PD42) and late passage (PD220) hTERT/E7 immortalized HFKs (Figure 6D). Together, these data indicate that Bmi1 protein is not only increased acutely by hTERT in primary keratinocytes but that its increased expression is maintained in late passage immortalized cells.

### Bmi1 cooperates with HPV E7 and immortalizes primary keratinocytes

**Figure 7:**
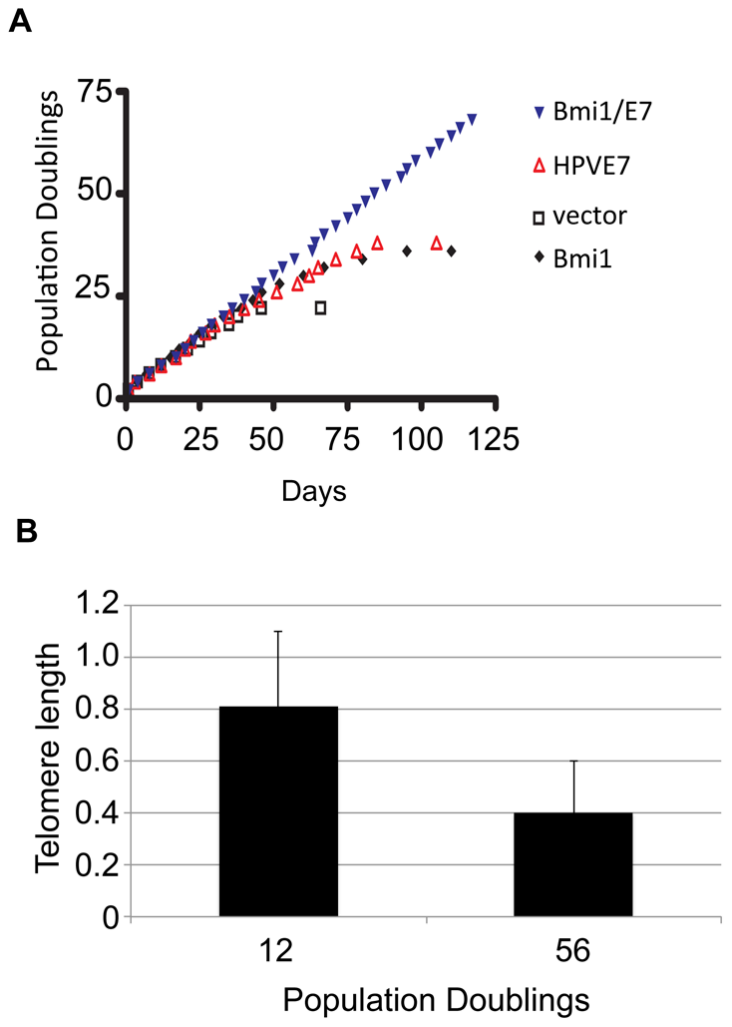
Bmi1 cooperates with E7 in cell immortalization. HFKs were doubly infected with pX-Bmi1 and pLXSN-E7, pLXSN-E7 alone, or empty vector. (A) Growth curve. Cells were passaged as described in the Methods section to determine the growth rate and lifespan of the cell populations. Bmi1, in cooperation with E7, induced cell immortalization equivalent to E6 or hTERT and E7. (B) Telomere length. A quantitative PCR-based technique (see Methods) was used to quantify the average telomere length in the indicated cell cultures.

Given the correlative data between hTERT and Bmi1 expression and a previous study showing that Bmi1 immortalizes mammary and oral epithelial cells [51], we speculated that Bmi1 might substitute for hTERT and immortalize human keratinocytes. To test this, we transduced HFKs with Bmi1 and E7 together or separately, as well as with empty vector. Cells were passaged to determine the growth rate and lifespan of these cell populations (**Figure 7A**). As expected, the HFKs infected with empty vector alone failed to reach 25 population doublings. Introducing E7 or Bmi1 alone extended lifespan by approximately 15 population doublings, as previously described for E7 [8]. However, cell immortalization (>50 population doublings) was observed only when Bmi1 and E7 were co-expressed (**Figure 7B**). It is noteworthy that the Bmi1/E7 cells also exhibited shortened telomere length, similar to E6/E7 cells. Thus, these data indicate that Bmi1, like hTERT and E6, is able to cooperate with E7 in the immortalization of HFKs.

### Bmi1 protein is increased in immortalized cervical cell lines and tumor cells

**Figure 8:**
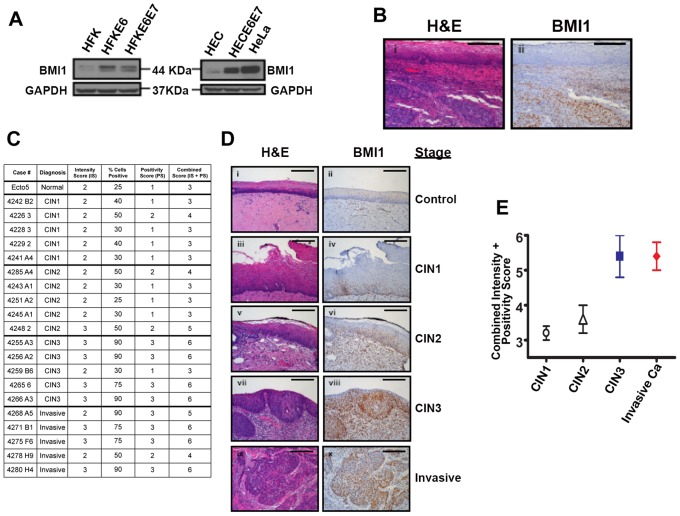
Bmi1 protein expression is increased in immortalized and tumorigenic cervical cell lines and positively correlates with disease stage in cervical dysplasia and neoplasia *in vivo*. (**A**) Bmi1 protein levels were quantified by Western blot in primary HFKs and primary human ectocervical cells (HECs) expressing E6 or immortalized by E6/E7 and the cervical cancer cell line HeLa. Lysates were separated by 4–20% gradient SDS-PAGE. Antibodies were used to detect hTERT (1∶1000, Origene), Bmi1 (1∶200, F6, Millipore) and GAPDH (1∶2000, FL-335, Santa Cruz). (**B**)Tissue from a case of invasive cervical cancer was acquired. Representative images are shown containing cancerous lesions and adjacent normal, intact epithelium. Tissue staining with hematoxylin and eosin (**i**) and immunohistochemical stain with Bmi1 (1∶200, F6, Millipore) (**ii**) are shown. (**C**)Tissues of Cervical Intraepithelial Neoplasia Stage 1 (CIN1), CIN2, CIN3 or carcinoma *in situ*, and invasive cervical carcinoma were acquired. To quantify Bmi1 expression, stained slides were subjected to a randomized, blinded review by a board-certified clinical pathologist. A subset of slides was scored multiple times to demonstrate reproducibility. For each sample, the case number and diagnosis is provided with the corresponding an intensity score, the percentage of positive cells, the corresponding positivity score, and the combined score. Each case received an intensity score from 0–3 (0 = negative, 1 = weak, 2 = moderate, 3 = intense) and the percentage of positive cells was recorded, which was converted to a positivity score (0 = less than 10%, 1 = 11–49%, 2 = 50–74%, 3 = 75–100%). Combined scores were calculated by adding the intensity score and positivity scores. (**D**) Immunohistochemical staining with hematoxylin and eosin (**i, iii, v, vii, ix**) and for Bmi1 protein (1∶100, F6, Millipore) (**ii, iv, vi, viii, x**) was performed. Representative images are shown. Relevant controls are shown, staining with hematoxylin and eosin (**i**) and for Bmi1 protein (1∶100, F6, Millipore) (**ii**). Scale bar = 50 µm. (**E**) Mean and standard deviation of combined scores are shown.

Although Bmi1 was increased in immortalized, non-tumorigenic HFKs, we queried whether its expression might be altered in cervical cells and during the progression to cervical cancer. We therefore first examined Bmi1 expression in immortalized and tumorgenic cervical cell lines. Consistent with our data in HFKs, expression of E6/E7 in primary human ectocervical cells (HECs) leads to immortalization, enhances endogenous hTERT expression, and increases in both Bmi1 mRNA and protein (**Figure 8A** **, Figure S6**). Bmi1 protein levels also showed increases in the tumor-derived, telomerase-positive HeLa cancer cell line (**Figure 8A** **,** **1A**). These data indicated that Bmi1 is increased in HPV-immortalized and tumorigenic cervical cells. Furthermore, immunohistochemical (IHC) staining of cervical cancer tissue specimens demonstrated increased Bmi1 levels in invasive lesions (**Figure 8B**).

### Bmi1 expression is highest in CIN3 and invasive carcinoma

Given the evidence demonstrating increased expression of Bmi1 in immortalized cells and tumorigenic cervical cancer cells and the observation that even invasive cervical tumors overexpress Bmi1, we examined whether Bmi1 expression correlated with the severity of cervical cancer progression. Bmi1 levels were evaluated by IHC in 21 cervical tissues identified by pathological review as either normal, precancerous, or cancerous lesions. Bmi1 was observed in modest amounts in the nucleus of cells in the basal and immediate suprabasal epithelium in normal cervical tissue (**Figure 8D** **ii**), CIN1, and CIN2 (**Figure 8D** **iv, vi**). However, a striking increase in staining was observed in the epithelial layers of CIN3 and invasive carcinoma (**Figure 8D** **viii, x**). For cases of invasive cervical carcinoma, Bmi1 staining was specific to cancerous lesions (**Figure 8D** **x**). To quantify Bmi1 expression, intensity and positivity scores were determined (**Figure 8C**) with the mean and standard deviation shown (**Figure 8E**). CIN3 and invasive carcinoma show combined scores that are significantly higher than both CIN1 and CIN2 (CIN3 vs. CIN1, *p* = 0.0083; CIN3 vs. CIN2, *p* = 0.0372; invasive vs. CIN1, *p* = 0.0012; invasive vs. CIN2, *p* = 0.0130; p values as calculated by student t-test). These data indicate that Bmi1 expression does indeed correlate with the degree of cervical dysplasia and with progression to cancer.

## Discussion

Overall our findings demonstrate that a novel extra-telomeric and non-catalytic function(s) of hTERT contributes to cell immortalization by hTERT and E7 in human keratinocytes. These findings not only define new properties of hTERT that contribute to cell immortalization, but they potentially modify our concept of the mechanism by which E6 is mediating cell immortalization. Using our current and published data, we have constructed a summary table listing the properties of HFK expressing the various hTERT mutants and HPV oncogenes (**Table S1**).

While we have shown that E6 and E7 are required for the efficient cell immortalization of primary cells, one study has shown that E7 immortalizes human keratinocytes at a very low efficiency when cultured in serum-free synthetic medium [8], [52] and another study has shown that wild type HPV16 E6 as well as an natural mutant E6 are able to immortalize human keratinocytes [53]. However, it is important to note that when E6 and E7 are used alone, an obvious “crisis” period or “flat” phase of cell growth is observed, indicating that cell immortalization is infrequent and arises from a small subpopulation of cells. Most likely additional genetic or epigenetic changes are required for escape from “crisis”. Although we have also noted that E7 is highly efficient for immortalizing keratinocytes without a “crisis” period of time when co-cultured with feeders or conditioned medium (Liu, X., et al. unpublished data), this is best explained by the ability of feeder cells or conditioned medium to induce telomerase [54].

A basic tenet of cell immortalization is that hTERT reverse transcriptase activity is essential for maintaining or elongating telomeres, thus allowing for continued cell replication [11]. However, our early studies showed that E6-induced telomerase activity could be dissociated from telomere maintenance [10]. Supporting this hypothesis, the current study clearly indicates that immortalized cells exhibit similar levels of telomerase activity, yet telomeres shorten during cell passaging and stabilize at late passages (**Figure 1B, 1C**). We have also demonstrated the same pattern of telomere length in keratinocytes immortalized by a Rho kinase (ROCK) inhibitor [55], [56], [57]. These data suggest that extra-telomeric functions of telomerase or hTERT play a role during cell immortalization independent of telomere maintenance.

Several reports indicate that hTERT-HA fails to elongate telomeres and immortalize human fibroblasts [41], [42] or HA1 cells [40] despite a high level of telomerase activity. Surprisingly, we found that hTERT-HA reproducibly and efficiently immortalized HFK cells in cooperation with HPV E7 [26]. Even more interesting, these immortalized HFK cells had short telomeres (**Figure 2B**). We also observed the same results with the hTERT N+T mutant, which is positive for telomerase activity, but negative for telomere recruitment and elongation (**Figure 3**). Here, we demonstrate in keratinocytes that telomerase activity is not required, as the catalytically-defective hTERT-D868A retains its ability to immortalize HFKs in cooperation with E7.

We have also shown that immortalization is independent of the telomere lengthening function of hTERT. Telomere lengthening is predominantly carried out by telomerase, but can also occur via the alternative telomere lengthening (ALT) pathway [58]. However, the exact mechanisms of telomere maintenance or elongation remain elusive. Studies have suggested that many mechanisms, including enzymatic activity, telomere-capping, and recombination, may play roles in the final stabilization of telomeres in immortalized and human cancer cells [42], [58], [59], [60], [61], [62], [63].

A number of non-canonical functions for hTERT have been reported in literature, and the list is increasing rapidly [27], [28], [29], [30], [31], [33], [34], [47], [48], [64]. This led us to pursue whole genome expression studies to probe altered signaling pathways in primary cells expressing hTERT. Surprisingly, our data have shown that 88% of the genes altered by wild-type hTERT are also altered in the same direction by hTERT-D868A (**Figure 5B**). Somewhat surprisingly, hTERT-D868A regulates about twice as many genes as wild-type hTERT, suggesting that elimination of the catalytic function of hTERT actually augments non-canonical functions.

It is critical to note that bypass of the Hayflick limit by enzymatic-defective hTERT mutants is accompanied by the global induction of many cellular genes, including endogenous hTERT (**Figure 3C**). This induction of endogenous hTERT is not due to the direct, acute transactivation of endogenous hTERT by mutant hTERT (**Figure 3A** **and** **4A**). Rather the endogenous hTERT activation is part of the larger number of gene sets that are increased during transit through M1/M2 restriction points.

A very recent study [47] demonstrates the existence of a splice-variant of hTERT (Δ4-13) containing an in-frame deletion of exons 4 through 13 that encode the catalytic domain of telomerase. This variant is expressed in telomerase negative normal cells and tissues as well as in transformed telomerase positive cell lines and in cells that employ an alternative method to maintain telomere length. The overexpression of the Δ4-13 significantly elevated the proliferation rate of several cell types without enhancing telomerase activity, while decreasing the endogenous expression of this variant using siRNA technology reduced cell proliferation. The expression of the Δ4-13 variant stimulates Wnt signaling. This is the first report that a naturally occurring hTERT splice variant that lacks telomerase activity exhibits an ability to stimulate cell proliferation, supporting our conclusions that non-canonical hTERT functions contribute cell immortalization.

We have also used real-time RT-PCR to validate more than 20 genes that were altered greater than two-fold as determined by microarray analysis. The RT-PCR results were virtually identical to the microarray data, and several of these genes are critical regulators of keratinocyte growth, apoptosis, and differentiation. Bmi1 was one such target of hTERT. Our microarray data also demonstrated that E6 and hTERT increased the expression of RB and ROCK1 mRNA in HFKs (**Dataset S2**) and this increased expression was confirmed by real time RT-PCR. This induction of RB is presumably counteracted by the activity of the E7 protein. There are similar parallel events in the cooperation of the E6 and E7 proteins in cell immortalization and transformation [6], [7], [8]. For example, these two genes seem to have evolved both complementary and opposing functions that are necessary to prevent senescence and/or apoptosis. For example, while E7 stabilizes p53 protein, E6 degrades this tumor suppressor protein. Similarly, while E6 stabilizes RB protein, E7 inactivates and destabilizes it. The yin-yang regulation of E6/E7 functions and telomerase and RB/16 pathways may be critical for fine tuning the growth and differentiation of keratinocytes as well as for regulating the viral replication cycle.

Bmi1 has been identified as a marker of cancer progression in a number of carcinomas, including those derived from the nasopharynx, breast, pancreas, and other sites [65], [66], [67]. Equally important, hTERT has been identified a potential universal cancer target, since it is up-regulated in most cancers [15], [68]. Beyond identification of Bmi1 in our genetic screen, hTERT and Bmi1 have been linked in several previous reports. PcG components Bmi1 and SIRT1 have been shown to be altered in hTERT-expressing urothelial cells [45], and Bmi1 has been shown to induce endogenous telomerase in human mammary epithelial cells [35]. Besides Bmi1, other chromatin remodeling complex members have also been associated with the non-telomere effects of hTERT, including transcriptional regulation of Wnt targets by binding BRG1, a Trithorax group protein (TrxG) [30]. Our data links hTERT expression to changes in Bmi1, suggesting that there is an hTERT-Bmi1 signaling pathway. In this study, we have shown that Bmi1 overexpression occurs prior to full transformation since both hTERT-expressing HFKs and multiple types of telomerase-positive immortalized cells (**Figure 5E** **,** **Figure 6** **, &** **Figure 8A**) overexpress Bmi1.

The above Bmi1 findings may have clinical relevance. Our *in vivo* studies reveal a differential expression of Bmi1 in carcinoma *in situ* and invasive carcinomas compared to preneoplastic lesions (**Figure 8B–E**). Indeed, Bmi1 mRNA expression is increased in cervical cancer compared to corresponding noncancerous tissues [69]. Additionally, Bmi1 overexpression has been significantly correlated with tumor size, clinical stage, and regional lymph node metastases in cancers of the cervix [70]. Another PcG protein, EZH2, was also recently shown to be up-regulated in high grade squamous cervical intraepithelial lesions (HSILs) compared to normal cervical epithelium [71], further implicating chromatin remodeling changes in tumor initiation and progression.

While Bmi1 appears to be a significant contributor to cell immortalization, it is also obvious that other genes detected in the mRNA expression screen may also contribute to this process. Indeed, the known anti-apoptotic activity of hTERT might also be expected to assist in the bypass of cellular senescence.

## Materials and Methods

### Plasmids and retroviruses

pLXSN vector and pLXSN-16E6, pLXSN-16E7, pLXSN-16E6E7 were as described previously [10], [21], [22], [72]. pBABE-puro-hTERT, pBABE-puro-hTERT-N+T [42], pBABE-puro-hTERT-D868A [43] were gifts from Dr. Elizabeth Blackburn, pBABE-puro-hTERT-HA [40] from Dr. Robert Weinberg, and pCLMSCV-puro-Bmi1 [36] from Dr. Tohru Kiyono. Other hTERT mutants were made using the QuikChange XL Site-Directed Mutagenesis Kit (Stratagene, La Jolla, CA). An N-terminal double Flag epitope tag was added to hTERT using a PCR insertion method. SD3443 retrovirus packaging cells were transfected with pLXSN vectors or pBABE-puro vectors described above using LipofectAmine 2000 (Invitrogen) as instructed. Culture supernatants containing retrovirus were collected 48 hours after transfection.

### Cell culture and generation of stable cell lines

Primary human foreskin keratinocytes (HFKs) and human foreskin fibroblasts (HFFs) were isolated and cultured from neonatal foreskins as described58. Primary human ectocervical keratinocytes (HECs) were derived from fresh cervical tissue similarly and obtained after hysterectomy for benign uterine diseases. Standard trypsinization procedures were used to isolate the keratinocytes, which were cultured in serum-free keratinocyte medium supplemented with 50 µg/ml of bovine pituitary extract and 25 ng/ml of recombinant epidermal growth factor (Invitrogen). The cells were cultured in serum-free keratinocyte growth media (Invitrogen) supplemented with gentamycin (50 µg/ml). Primary HFKs, HFFs, and HECs were transduced with amphotropic pLXSN retroviruses expressing HPV-16E6, E7, or both E6 and E7 and/or pBABE-puro retroviruses expressing hTERT or its mutants (see above). Retrovirus-transduced cells were selected in G418 (100 µg/ml) for 5 days and/or puromycin (2 ug/ml) for 3 days. Resistant colonies were pooled and passaged every 3–4 days (1∶4 ratio for HFKs and HECs, 1∶8 ratio for HFFs). HeLa, C33A, SiHa cells were maintained in complete DMEM medium. All cells were cultured on plastic tissue culture dishes or flasks.

### Formalin fixation and paraffin embedding

To prepare cells for immunocytochemistry, cells were pelleted and then fixed with 4% paraformaldehyde solution overnight and resuspended in HistoGel (Richard-Allan Scientific) at a ratio of 1∶1 per volume. The gel matrix was processed through graduated alcohols and Clear-Rite 3 (Richard-Allan Scientific) for paraffin embedding using the Leica ASP300 system (Leica Microsystems, Wetzlar, Germany). Paraffin sections were cut at 5 µm and mounted on Superfrost Plus slides (Fisher Scientific).

### Tissue

Patient samples were acquired through the Histopathology and Tissue Shared Resource at the Lombardi Comprehensive Cancer Center (Washington, DC). Twenty one cervical tissues were acquired which represented different pathological stages–one normal tissue core and five tissue cores for each of the following pathological stages: Cervical Intraepithelial Neoplasia Stage 1 (CIN1), CIN2, CIN3 or carcinoma *in situ*, and invasive cervical carcinoma.

### Real-time quantitative telomeric repeat amplification protocol (Q-TRAP)

Human keratinocytes and fibroblasts were lysed and analyzed by Q-TRAP [72] with SYBR Green Supermixture (Bio-Rad). A standard curve was produced for the real-time Q-TRAP assay using serially diluted HeLa cell extracts. All samples were run in triplicate.

### Telomere length

Genomic DNA was extracted from cells using Qiagen DNeasy Blood & Tissue Kit. Average telomere length was assessed by a modified method of the real-time PCR–based telomere assay [73]. Briefly, the telomere repeat copy number to single gene copy number (T/S) ratio was determined using the Bio-Rad IQ5 thermocycler in a 96-well format. Five nanograms of genomic DNA was subjected to PCR reactions with Bio-Rad SYBR Green Super mixture. The primers for telomere length and HBG1 (a single copy gene) were as below:

Tel-1 5′ CGGTTTGTTTGGGTTTGGGTTTGGGTTTGGGTTTGGGTT-3,

Tel-2 5′-GGCTTGCCTTACCCTTACCCTTACCCTTACCCTTACCCT-3′;

HBG1 5′- TGTGCTGGCCCATCACTTTG,

HBG2 5′- ACCAGCCACCACTTTCTGATAGG-3′.

The reactions proceeded for 1 cycle at 95°C for 5 min, followed by 41 cycles at 95°C for 15 s, 60°C for 45 s. All samples for both the telomere and HBG1 reactions were done in triplicate. In addition to the samples, each 96-well plate contained a six-point standard curve from 0.0, 0.2, 1.0, 5.0, 25.0, 125.0 ng using genomic DNA (telomere length 10.4 kb) from Roche Telo-kit. The T/S ratio (dCt) for each sample was calculated by normalizing the average HBG1 Ct value from the average telomere Ct value.

### Luciferase assay

1×10^5^ telomerase-negative HFKs were seeded onto 24-well plates and grown overnight. Transient transfections were performed using LipofectAmine 2000 reagent (Invitrogen) according to the protocol provided by the manufacturer. Cotransfections were performed using 0.5 ug of a core hTERT or Cyclin D1 promoter reporter plasmids and 50 ng of each expression vector as indicated (HPV16E6, hTERTwt or hTERT-D868A) or empty vectors as control for basal promoter activity. Cells also were cotransfected with 2 ng of the pRL-CMV plasmid (Promega), which contains the Renilla reniformis luciferase gene as a transfection control. Firefly and Renilla luciferase activities were measured 24 hr after transfection using the Dual luciferase reporter assay system (Promega).

### Microarray

hTERT, hTERT-D868A, HPV E6 or the pBP vector was stably expressed in primary HFKs. Cells were grown on 100 mm tissue culture dishes (BD Falcon) to confluency before harvesting RNA with 1 mL TRIzol Reagent according to manufacturer's protocol. DNAse treatment was performed (Ambion, Austin, TX). RNA was sent to MOGene, LC (St. Louis, MO) for microarray analysis. E6, hTERT, and hTERT-D8686A were run separately against the pBP on a two-color Agilent whole human genome slide with a 4 x 44K format. A total of six comparative arrays were run- hTERT, hTERT-D868A, or E6 vs. empty vector and run with a duplicate for dye swap. RNA was amplified using the Agilent Low Input Linear Amplification kit (Agilent Technologies, Santa Clara, CA), and then labeled with either cyanine-5 or cyanine-3 using the ULS RNA Fluorescent Labeling Kit (Kreatech Biotechnology, Amsterdam, The Netherlands). 825 ng each of labeled c-DNA was hybridized overnight at 65°C in an ozone-free room to protect the label. All washes and hybridization conditions followed were consistent with the Agilent processing manual (protocol version 4.0). Arrays were scanned using an Agilent C scanner and extracted using the Agilent Feature Extraction software 10.7.1 (Agilent Technologies, Santa Clara, CA). Initial data analysis was performed by MOGene using the Rosetta Luminator software (Agilent). Expression arrays were submitted to DAVID Bioinformatics Resources 6.7 (NIAID, NIH) for Functional Annotation Clustering [49]. Using the MultiExperiment Viewer v4.8 (TM4 Microarray Software Suite, Rockville, MD) and data from the four hTERT comparative arrays, a heat map was constructed with the cluster of interest [50].

### cDNA and Quantitative Real Time PCR

SuperScript III Reverse Transcriptase kit (Invitrogen) was used to perform reverse transcription PCR (RT-PCR), as previously described [72]. Reactions were annealed and analyzed using a Bio-Rad iCycler and accompanying software (Bio-Rad Laboratories). Primer sets used include the following:

Bmi1-F: 5′ TGCCCAGCAGCAATGACTGT3′

Bmi1-R: 5′ GTCCATCTCTCTGGTGACTGATCTTC3′

GAPDH-F: 5′ TCTCCTCTGACTTCAACAGC3′

GAPDH-R: 5′ GAAATGAGCTTGACAAAGTG3′

### Western blots

Stable cell lines were lysed in 2X SDS gel electrophoresis sample buffer. Proteins were separated on a 4–20% Tris-glycine gradient gel (Invitrogen) and electrophoretically transferred to an Immobilon-P PVDF membrane (Millipore). The membranes were blocked in 5% dry milk-PBST and incubated with pRb antibody (1∶1000, Cell Signaling), hTERT (1∶1000, Y182, Epitomics), Bmi1 (1∶200, F6, Millipore), P53 (1∶1000, Pab 1801, Santa Cruz), and HPV16-E7 (1∶1000, ED17, Santa Cruz); and a secondary antibody with HRP conjugation and detected by chemiluminescence (anti-rabbit IgG or anti-mouse IgG; Santa Cruz Biotechnology). Equal protein sample loading was monitored using an anti-β-actin (1∶5000, Sigma) or anti-GAPDH (1∶2000, FL-335, Santa Cruz). The membranes were visualized by using Western Blotting Chemiluminescence Luminol Reagent (Santa Cruz).

### Immunocytochemistry and immunohistochemistry

Immunocytochemistry of HFK cell pellets and immunohistochemistry of cervical tissue was performed for Bmi1. Five micron sections from formalin fixed, paraffin embedded tissues were de-paraffinized with xylenes and rehydrated through a graded alcohol series. Heat induced epitope retrieval (HIER) was performed by immersing the tissue sections at 98°C for 20 minutes in 10 mM citrate buffer (pH 6.0) with 0.05% Tween. Immunohistochemical staining was performed using the VectaStain Kit from Vector Labs according to manufacturer's instructions. Briefly, slides were treated with 3% hydrogen peroxide for 10 minutes. Endogenous biotin was blocked using an avidin/biotin blocking kit from Invitrogen. The slides were then treated with 10% normal goat serum and exposed to primary antibodies for Bmi1 (1∶200, F6, Millipore) for 1 hour at 22°C. Slides were exposed to appropriate biotin-conjugated secondary antibodies (Vector Labs), Vectastain ABC reagent and DAB chromagen (Dako). Slides were counterstained with Hematoxylin (Fisher, Harris Modified Hematoxylin) at a 1∶17 dilution for 2 minutes at RT, blued in 1% ammonium hydroxide for 1 minute at 22°C, dehydrated, and mounted with Acrymount. Consecutive sections with the omitted primary antibody were used as negative controls.

### Immunohistochemistry scoring

To quantify expression of immunohistochemical staining, slides were subjected to a randomized, blinded scoring performed by a board-certified clinical pathologist. Combined scores were calculated by adding the intensity score and positivity scores. Mean and standard deviation of combined scores were calculated. A subset of slides was scored multiple times to demonstrate reproducibility. Each case received an intensity score from 0–3 (0 = negative, 1 = weak, 2 = moderate, 3 = intense) and the percentage of positive cells was recorded, which was converted to a tiered positivity score (0 = less than 10%, 1 = 11–49%, 2 = 50–74%, 3 = 75–100%).

### Immunofluorescence microscopy

HFKs were transfected with either wild-type hTERT or an hTERT mutant, and then grown on sterile glass cover slips, fixed in 4% (wt/vol) paraformaldehyde, and labeled with the primary and secondary antibodies. The following primary antibodies were used: anti-hTERT (Rockland 1∶500 dilution) and anti-hTERT serum from rabbit immunized with KLH conjugated Ac-CSRKLPGTTLTALEAAANPAL-amide (aa1104-1123). The secondary antibodies, AlexaFluor 488 donkey anti-mouse IgG and AlexaFluor 555 donkey anti-rabbit IgG (Invitrogen) were used at a concentration of 5 µg/mL. A Zeiss Axioskop microscope and a HAMAMATSU ORCA-ER Digital Camera were used for visualization and microphotography.

### Ethics statement

The HFK cells were prepared from human neonatal foreskins at Georgetown University Hospital, normally these tissues are de-identified and discarded. The cervical tissue samples from the Histopathology and Tissue Shared Resource at the Lombardi Comprehensive Cancer Center were anonymized. These protocols (2002-021 and 1992-048) have been approved by the Georgetown University Institutional Review Board.

### Gene list

BMI1 (NM_005180), BRG1 (NM_003072), EGFR (NM_005228), EZH2 (NM_004456), FGF2 (NM_002006), HPV16 E6 (NP_041325), HPV16 E7 (NP_041326), hTERT (NM_198253), P16 (NM_058195), RB (NM_000321), SIRT1 (NM_012238), TP53 (NM_000546), VEGF (NM_003376).

## Supporting Information

Dataset S1 **E6, hTERTwt, and hTERT-D868A alter the expression of overlapping gene sets.** Consolidated microarray data showing significant changes from all six hybridizations, with lists of overlapping gene sets, as described in Figure 6A–C.
(XLSX)

Dataset S2 **hTERT alters chromatin remodeling genes, including Bmi1.** Following identification of the 2077 hTERT RT-independent gene changes (changes shared by hTERTwt and hTERT-D868A as shown in Figure 6B), arrays were submitted for functional annotation clustering. Of the clusters identified, one of the top ten clusters formed based on enrichment scores was a cluster of 91 probes related to chromatin remodeling, shown as a list.
(XLSX)

Figure S1 **pRb levels validate the expression of a functional E7 protein.** Cell lysates were extracted with 2x SDS buffer and subjected to SDS-PAGE gel and blotted with anti-Rb antibody. β-actin was used as internal control. pRb level decrease in E7, hTERT/E7 and hTERT-HA/E7 expressing cells.
(TIF)

Figure S2 **Wild-type and hTERT mutants localize to the nucleus.** The hTERT-D868A mutant expresses and localizes in the nucleus similar to wild-type hTERT.
(TIF)

Figure S3 **Scheme of array-based whole genome expression analysis.** (A) We stably expressed E6, hTERT wt or a catalytically inactive mutant hTERT (D868A) in primary HFKs. Cells were lysed using the TRIzol reagent and RNA isolated following the manufacturer's protocol (Invitrogen) from samples 12–14 days post-infection. RNA quality was assessed by bioanalyzer. RNA was reverse transcribed and labeled with fluorescent dyes (Cy3 or Cy5) and submitted for array analysis using the Agilent 4 x 44K format. (B) Dye swap comparisons were made and directionally consistent changes were identified. The high percentage of consistency in expression changes are shown in the chart and support the integrity of the data.
(TIF)

Figure S4 **Visual representation of chromatin remodeling genes altered by hTERT.** After identification of the chromatin cluster through functional annotation analysis, a heat map visualizing this cluster of probes was constructed. The four TERT arrays (two arrays for hTERT wt, two arrays for hTERT-D868A, duplicate samples with dye swap). Red represents a decrease in fold change vs empty vector (ev) while green represents an increase in fold change vs ev, and black represents no change. Intensity of color correlates to intensity of fold change, as described by the scale bar. Of the ninety one probes, Bmi1 is highlighted.
(TIF)

Figure S5 **Bmi1 mRNA and Bmi1 protein increase with the expression of inactive hTERT mutants.** To further validate Bmi1 increases seen by hTERT wt and hTERT-D868A expressing cells, additional hTERT mutants were also tested to determine if their expression increased Bmi1 mRNA levels. (A) Quantitative RT-PCR was performed with gene-specific primers for Bmi1, normalized to GAPDH. n = 3. Bars represent mean ± SD. 2F represents a double FLAG epitope tag on the N-terminus of the hTERT protein. Mutant AA1 had two leucine to alanine point mutations made at residues 837 and 840 (L837A, L840A) while AA2 had two leucine to alanine point mutations made at residues 863 and 866 (L863A, L866A). (B) Western blot confirmation of hTERT expression using a FLAG antibody for detection (1∶1000, Sigma). Samples were run on the same 4–20% gradient SDS-PAGE but separated by several lanes. Image cropped accordingly. (C) Quantitative TRAP confirms positive TRAP activity in hTERT wt and suggests mutants AA1 and AA2 are TRAP negative, compared to empty vector alone.
(TIF)

Figure S6 **Bmi1 mRNA is increased in E6/E7 HFKs compared to empty vector alone.** Quantitative RT-PCR was performed on empty vector and E6/E7 HFKs with gene-specific primers for Bmi1, normalized to GAPDH. n = 3. Bars represent mean ± SD.
(TIF)

Table S1 **Properties of cells immortalized by hTERT, E6, and E7.**
(PDF)

## References

[1] Hanahan D, Weinberg RA (2011) Hallmarks of cancer: the next generation. Cell 144: 646–674. 21376230 10.1016/j.cell.2011.02.013

[2] Hawley-Nelson P, Vousden KH, Hubbert NL, Lowy DR, Schiller JT (1989) HPV16 E6 and E7 proteins cooperate to immortalize human foreskin keratinocytes. Embo J 8: 3905–3910. 2555178 10.1002/j.1460-2075.1989.tb08570.x PMC402081

[3] Munger K, Phelps WC, Bubb V, Howley PM, Schlegel R (1989) The E6 and E7 genes of the human papillomavirus type 16 together are necessary and sufficient for transformation of primary human keratinocytes. J Virol 63: 4417–4421. 2476573 10.1128/jvi.63.10.4417-4421.1989 PMC251060

[4] Schwarz E, Freese UK, Gissmann L, Mayer W, Roggenbuck B, et al (1985) Structure and transcription of human papillomavirus sequences in cervical carcinoma cells. Nature 314: 111–114. 2983228 10.1038/314111a0

[5] Munger K, Howley PM (2002) Human papillomavirus immortalization and transformation functions. Virus Res 89: 213–228. 12445661 10.1016/s0168-1702(02)00190-9

[6] Howie HL, Katzenellenbogen RA, Galloway DA (2009) Papillomavirus E6 proteins. Virology 384: 324–334. 19081593 10.1016/j.virol.2008.11.017 PMC2674106

[7] McLaughlin-Drubin ME, Munger K (2009) The human papillomavirus E7 oncoprotein. Virology 384: 335–344. 19007963 10.1016/j.virol.2008.10.006 PMC2661820

[8] Klingelhutz AJ, Roman A (2012) Cellular transformation by human papillomaviruses: lessons learned by comparing high- and low-risk viruses. Virology 424: 77–98. 22284986 10.1016/j.virol.2011.12.018 PMC3703738

[9] Klingelhutz AJ, Foster SA, McDougall JK (1996) Telomerase activation by the E6 gene product of human papillomavirus type 16. Nature 380: 79–82. 8598912 10.1038/380079a0

[10] Stoppler H, Hartmann DP, Sherman L, Schlegel R (1997) The human papillomavirus type 16 E6 and E7 oncoproteins dissociate cellular telomerase activity from the maintenance of telomere length. J Biol Chem 272: 13332–13337. 9148955 10.1074/jbc.272.20.13332

[11] Greider CW (1996) Telomere length regulation. Annu Rev Biochem 65: 337–365. 8811183 10.1146/annurev.bi.65.070196.002005

[12] Harley CB, Futcher AB, Greider CW (1990) Telomeres shorten during ageing of human fibroblasts. Nature 345: 458–460. 2342578 10.1038/345458a0

[13] Vaziri H, Schachter F, Uchida I, Wei L, Zhu X, et al (1993) Loss of telomeric DNA during aging of normal and trisomy 21 human lymphocytes. Am J Hum Genet 52: 661–667. 8460632 PMC1682068

[14] Counter CM, Avilion AA, LeFeuvre CE, Stewart NG, Greider CW, et al (1992) Telomere shortening associated with chromosome instability is arrested in immortal cells which express telomerase activity. Embo J 11: 1921–1929. 1582420 10.1002/j.1460-2075.1992.tb05245.x PMC556651

[15] Kim NW, Piatyszek MA, Prowse KR, Harley CB, West MD, et al (1994) Specific association of human telomerase activity with immortal cells and cancer. Science 266: 2011–2015. 7605428 10.1126/science.7605428

[16] Cukusic A, Skrobot Vidacek N, Sopta M, Rubelj I (2008) Telomerase regulation at the crossroads of cell fate. Cytogenet Genome Res 122: 263–272. 19188695 10.1159/000167812

[17] Kiyono T, Foster SA, Koop JI, McDougall JK, Galloway DA, et al (1998) Both Rb/p16INK4a inactivation and telomerase activity are required to immortalize human epithelial cells. Nature 396: 84–88. 9817205 10.1038/23962

[18] Natarajan E, Omobono JD 2nd, Guo Z, Hopkinson S, Lazar AJ, et al (2006) A keratinocyte hypermotility/growth-arrest response involving laminin 5 and p16INK4A activated in wound healing and senescence. Am J Pathol 168: 1821–1837. 16723698 10.2353/ajpath.2006.051027 PMC1606631

[19] Rheinwald JG, Hahn WC, Ramsey MR, Wu JY, Guo Z, et al (2002) A two-stage, p16(INK4A)- and p53-dependent keratinocyte senescence mechanism that limits replicative potential independent of telomere status. Mol Cell Biol 22: 5157–5172. 12077343 10.1128/MCB.22.14.5157-5172.2002 PMC139780

[20] Utikal J, Polo JM, Stadtfeld M, Maherali N, Kulalert W, et al (2009) Immortalization eliminates a roadblock during cellular reprogramming into iPS cells. Nature 460: 1145–1148. 19668190 10.1038/nature08285 PMC3987892

[21] Liu X, Dakic A, Chen R, Disbrow GL, Zhang Y, et al (2008) Cell-restricted immortalization by human papillomavirus correlates with telomerase activation and engagement of the hTERT promoter by Myc. J Virol 82: 11568–11576. 18818322 10.1128/JVI.01318-08 PMC2583678

[22] Liu X, Yuan H, Fu B, Disbrow GL, Apolinario T, et al (2005) The E6AP ubiquitin ligase is required for transactivation of the hTERT promoter by the human papillomavirus E6 oncoprotein. J Biol Chem 280: 10807–10816. 15655249 10.1074/jbc.M410343200

[23] James MA, Lee JH, Klingelhutz AJ (2006) HPV16-E6 associated hTERT promoter acetylation is E6AP dependent, increased in later passage cells and enhanced by loss of p300. Int J Cancer 119: 1878–1885. 16708385 10.1002/ijc.22064 PMC2223064

[24] Xu M, Luo W, Elzi DJ, Grandori C, Galloway DA (2008) NFX1 interacts with mSin3A/histone deacetylase to repress hTERT transcription in keratinocytes. Mol Cell Biol 28: 4819–4828. 18505829 10.1128/MCB.01969-07 PMC2493374

[25] Katzenellenbogen RA, Vliet-Gregg P, Xu M, Galloway DA (2009) NFX1-123 increases hTERT expression and telomerase activity post-transcriptionally in HPV 16E6 keratinocytes. J Virol 10.1128/JVI.02556-08 PMC2698580 19369336

[26] Liu X, Dakic A, Zhang Y, Dai Y, Chen R, et al (2009) HPV E6 protein interacts physically and functionally with the cellular telomerase complex. Proc Natl Acad Sci U S A 106: 18780–18785. 19843693 10.1073/pnas.0906357106 PMC2773972

[27] Rahman R, Latonen L, Wiman KG (2005) hTERT antagonizes p53-induced apoptosis independently of telomerase activity. Oncogene 24: 1320–1327. 15608686 10.1038/sj.onc.1208232

[28] Lee J, Sung YH, Cheong C, Choi YS, Jeon HK, et al (2008) TERT promotes cellular and organismal survival independently of telomerase activity. Oncogene 27: 3754–3760. 18223679 10.1038/sj.onc.1211037

[29] Artandi SE, Alson S, Tietze MK, Sharpless NE, Ye S, et al (2002) Constitutive telomerase expression promotes mammary carcinomas in aging mice. Proc Natl Acad Sci U S A 99: 8191–8196. 12034875 10.1073/pnas.112515399 PMC123043

[30] Park JI, Venteicher AS, Hong JY, Choi J, Jun S, et al (2009) Telomerase modulates Wnt signalling by association with target gene chromatin. Nature 460: 66–72. 19571879 10.1038/nature08137 PMC4349391

[31] Masutomi K, Possemato R, Wong JM, Currier JL, Tothova Z, et al (2005) The telomerase reverse transcriptase regulates chromatin state and DNA damage responses. Proc Natl Acad Sci U S A 102: 8222–8227. 15928077 10.1073/pnas.0503095102 PMC1149439

[32] Smith LL, Coller HA, Roberts JM (2003) Telomerase modulates expression of growth-controlling genes and enhances cell proliferation. Nat Cell Biol 5: 474–479. 12717449 10.1038/ncb985

[33] Zhou L, Zheng D, Wang M, Cong YS (2009) Telomerase reverse transcriptase activates the expression of vascular endothelial growth factor independent of telomerase activity. Biochem Biophys Res Commun 386: 739–743. 19559675 10.1016/j.bbrc.2009.06.116

[34] Jin X, Beck S, Sohn YW, Kim JK, Kim SH, et al (2010) Human telomerase catalytic subunit (hTERT) suppresses p53-mediated anti-apoptotic response via induction of basic fibroblast growth factor. Exp Mol Med 42: 574–582. 20628269 10.3858/emm.2010.42.8.058 PMC2928930

[35] Dimri GP, Martinez JL, Jacobs JJ, Keblusek P, Itahana K, et al (2002) The Bmi-1 oncogene induces telomerase activity and immortalizes human mammary epithelial cells. Cancer Res 62: 4736–4745. 12183433

[36] Haga K, Ohno S, Yugawa T, Narisawa-Saito M, Fujita M, et al (2007) Efficient immortalization of primary human cells by p16INK4a-specific short hairpin RNA or Bmi-1, combined with introduction of hTERT. Cancer Sci 98: 147–154. 17233832 10.1111/j.1349-7006.2006.00373.x PMC11158394

[37] Sparmann A, van Lohuizen M (2006) Polycomb silencers control cell fate, development and cancer. Nat Rev Cancer 6: 846–856. 17060944 10.1038/nrc1991

[38] Simon JA, Kingston RE (2009) Mechanisms of polycomb gene silencing: knowns and unknowns. Nat Rev Mol Cell Biol 10: 697–708. 19738629 10.1038/nrm2763

[39] zur Hausen H (2009) Papillomaviruses in the causation of human cancers - a brief historical account. Virology 384: 260–265. 19135222 10.1016/j.virol.2008.11.046

[40] Counter CM, Hahn WC, Wei W, Caddle SD, Beijersbergen RL, et al (1998) Dissociation among in vitro telomerase activity, telomere maintenance, and cellular immortalization. Proc Natl Acad Sci U S A 95: 14723–14728. 9843956 10.1073/pnas.95.25.14723 PMC24516

[41] Ouellette MM, Aisner DL, Savre-Train I, Wright WE, Shay JW (1999) Telomerase activity does not always imply telomere maintenance. Biochem Biophys Res Commun 254: 795–803. 9920820 10.1006/bbrc.1998.0114

[42] Kim M, Xu L, Blackburn EH (2003) Catalytically active human telomerase mutants with allele-specific biological properties. Exp Cell Res 288: 277–287. 12915119 10.1016/s0014-4827(03)00217-9

[43] Zhu J, Wang H, Bishop JM, Blackburn EH (1999) Telomerase extends the lifespan of virus-transformed human cells without net telomere lengthening. Proc Natl Acad Sci U S A 96: 3723–3728. 10097104 10.1073/pnas.96.7.3723 PMC22361

[44] Armbruster BN, Etheridge KT, Broccoli D, Counter CM (2003) Putative telomere-recruiting domain in the catalytic subunit of human telomerase. Mol Cell Biol 23: 3237–3246. 12697823 10.1128/MCB.23.9.3237-3246.2003 PMC153184

[45] Chapman EJ, Kelly G, Knowles MA (2008) Genes involved in differentiation, stem cell renewal, and tumorigenesis are modulated in telomerase-immortalized human urothelial cells. Mol Cancer Res 6: 1154–1168. 18644980 10.1158/1541-7786.MCR-07-2168 PMC3437422

[46] Ghosh A, Saginc G, Leow SC, Khattar E, Shin EM, et al (2012) Telomerase directly regulates NF-kappaB-dependent transcription. Nat Cell Biol 14: 1270–1281. 23159929 10.1038/ncb2621

[47] Hrdlickova R, Nehyba J, Bose HR Jr (2012) Alternatively spliced TERT variants lacking telomerase activity stimulate cell proliferation. Mol Cell Biol 32: 4283–96. 22907755 10.1128/MCB.00550-12 PMC3486134

[48] Liu Z, Li Q, Li K, Chen L, Li W, et al (2012) Telomerase reverse transcriptase promotes epithelial-mesenchymal transition and stem cell-like traits in cancer cells. Oncogene doi:10.1038/onc.2012.441. [Epub ahead of print]. 10.1038/onc.2012.441 23045275

[49] Huang da W, Sherman BT, Lempicki RA (2009) Systematic and integrative analysis of large gene lists using DAVID bioinformatics resources. Nat Protoc 4: 44–57. 19131956 10.1038/nprot.2008.211

[50] Saeed AI, Sharov V, White J, Li J, Liang W, et al (2003) TM4: a free, open-source system for microarray data management and analysis. Biotechniques 34: 374–378. 12613259 10.2144/03342mt01

[51] Kim RH, Kang MK, Shin KH, Oo ZM, Han T, et al (2007) Bmi-1 cooperates with human papillomavirus type 16 E6 to immortalize normal human oral keratinocytes. Exp Cell Res 313: 462–472. 17161394 10.1016/j.yexcr.2006.10.025

[52] Halbert CL, Demers GW, Galloway DA (1991) The E7 gene of human papillomavirus type 16 is sufficient for immortalization of human epithelial cells. J Virol 65: 473–478. 1845902 10.1128/jvi.65.1.473-478.1991 PMC240541

[53] Niccoli S, Abraham S, Richard C, Zehbe I (2012) The Asian-American E6 variant protein of human papillomavirus 16 alone is sufficient to promote immortalization, transformation, and migration of primary human foreskin keratinocytes. J Virol 86: 12384–12396. 22951839 10.1128/JVI.01512-12 PMC3486486

[54] Fu B, Quintero J, Baker CC (2003) Keratinocyte growth conditions modulate telomerase expression, senescence, and immortalization by human papillomavirus type 16 E6 and E7 oncogenes. Cancer Res 63: 7815–7824. 14633708

[55] Chapman S, Liu X, Meyers C, Schlegel R, McBride AA (2010) Human keratinocytes are efficiently immortalized by a Rho kinase inhibitor. J Clin Invest 120: 2619–2626. 20516646 10.1172/JCI42297 PMC2898606

[56] Liu X, Ory V, Chapman S, Yuan H, Albanese C, et al (2012) ROCK inhibitor and feeder cells induce the conditional reprogramming of epithelial cells. Am J Pathol 180: 599–607. 22189618 10.1016/j.ajpath.2011.10.036 PMC3349876

[57] Suprynowicz FA, Upadhyay G, Krawczyk E, Kramer SC, Hebert JD, et al (2012) Conditionally reprogrammed cells represent a stem-like state of adult epithelial cells. Proc Natl Acad Sci U S A 109: 20035–20040. 23169653 10.1073/pnas.1213241109 PMC3523865

[58] Cesare AJ, Reddel RR (2010) Alternative lengthening of telomeres: models, mechanisms and implications. Nat Rev Genet 11: 319–330. 20351727 10.1038/nrg2763

[59] Royle NJ, Foxon J, Jeyapalan JN, Mendez-Bermudez A, Novo CL, et al (2008) Telomere length maintenance–an ALTernative mechanism. Cytogenet Genome Res 122: 281–291. 19188697 10.1159/000167814

[60] Yang Q (2008) Cellular senescence, telomere recombination and maintenance. Cytogenet Genome Res 122: 211–218. 19188689 10.1159/000167806

[61] Morrish TA, Greider CW (2009) Short telomeres initiate telomere recombination in primary and tumor cells. PLoS Genet 5: e1000357. 19180191 10.1371/journal.pgen.1000357 PMC2627939

[62] Svenson U, Roos G (2009) Telomere length as a biological marker in malignancy. Biochem Biophys Acta 1792: 317–323. 19419696 10.1016/j.bbadis.2009.01.017

[63] Strong MA, Vidal-Cardenas SL, Karim B, Yu H, Guo N, et al (2011) Phenotypes in mTERT(+)/(−) and mTERT(−)/(−) mice are due to short telomeres, not telomere-independent functions of telomerase reverse transcriptase. Mol Cell Biol 31: 2369–2379. 21464209 10.1128/MCB.05312-11 PMC3133422

[64] Parkinson EK, Fitchett C, Cereser B (2008) Dissecting the non-canonical functions of telomerase. Cytogenet Genome Res 122: 273–280. 19188696 10.1159/000167813

[65] Song LB, Zeng MS, Liao WT, Zhang L, Mo HY, et al (2006) Bmi-1 is a novel molecular marker of nasopharyngeal carcinoma progression and immortalizes primary human nasopharyngeal epithelial cells. Cancer Res 66: 6225–6232. 16778197 10.1158/0008-5472.CAN-06-0094

[66] Guo BH, Feng Y, Zhang R, Xu LH, Li MZ, et al (2011) Bmi-1 promotes invasion and metastasis, and its elevated expression is correlated with an advanced stage of breast cancer. Mol Cancer 10: 10. 21276221 10.1186/1476-4598-10-10 PMC3038148

[67] Song W, Tao K, Li H, Jin C, Song Z, et al (2010) Bmi-1 is related to proliferation, survival and poor prognosis in pancreatic cancer. Cancer Sci 101: 1754–1760. 20426791 10.1111/j.1349-7006.2010.01577.x PMC11159722

[68] Harley CB (2008) Telomerase and cancer therapeutics. Nat Rev Cancer 8: 167–179. 18256617 10.1038/nrc2275

[69] Min L, Dong-Xiang S, Xiao-Tong G, Ting G, Xiao-Dong C (2011) Clinicopathological and prognostic significance of Bmi-1 expression in human cervical cancer. Acta Obstet Gynecol Scand 90: 737–745. 21309753 10.1111/j.1600-0412.2011.01102.x

[70] Zhang X, Wang CX, Zhu CB, Zhang J, Kan SF, et al (2010) Overexpression of Bmi-1 in uterine cervical cancer: correlation with clinicopathology and prognosis. Int J Gynecol Cancer 20: 1597–1603. 21370603

[71] Hyland PL, McDade SS, McCloskey R, Dickson GJ, Arthur K, et al (2011) Evidence for alteration of EZH2, BMI1, and KDM6A and epigenetic reprogramming in human papillomavirus type 16 E6/E7-expressing keratinocytes. J Virol 85: 10999–11006. 21865393 10.1128/JVI.00160-11 PMC3194988

[72] Liu X, Roberts J, Dakic A, Zhang Y, Schlegel R (2008) HPV E7 contributes to the telomerase activity of immortalized and tumorigenic cells and augments E6-induced hTERT promoter function. Virology 375: 611–623. 18367227 10.1016/j.virol.2008.02.025 PMC2716003

[73] Cawthon RM (2009) Telomere length measurement by a novel monochrome multiplex quantitative PCR method. Nucleic Acids Res 37: e21. 19129229 10.1093/nar/gkn1027 PMC2647324

