# Supplementary figures and images for: HPV16 E7 Protein and hTERT Proteins Defective for Telomere Maintenance Cooperate to Immortalize Human Keratinocytes
Source: PLoS Pathog. 2013 Apr 4;9(4):e1003284. doi: 10.1371/journal.ppat.1003284 (PMC3617164; doi:10.1371/journal.ppat.1003284)

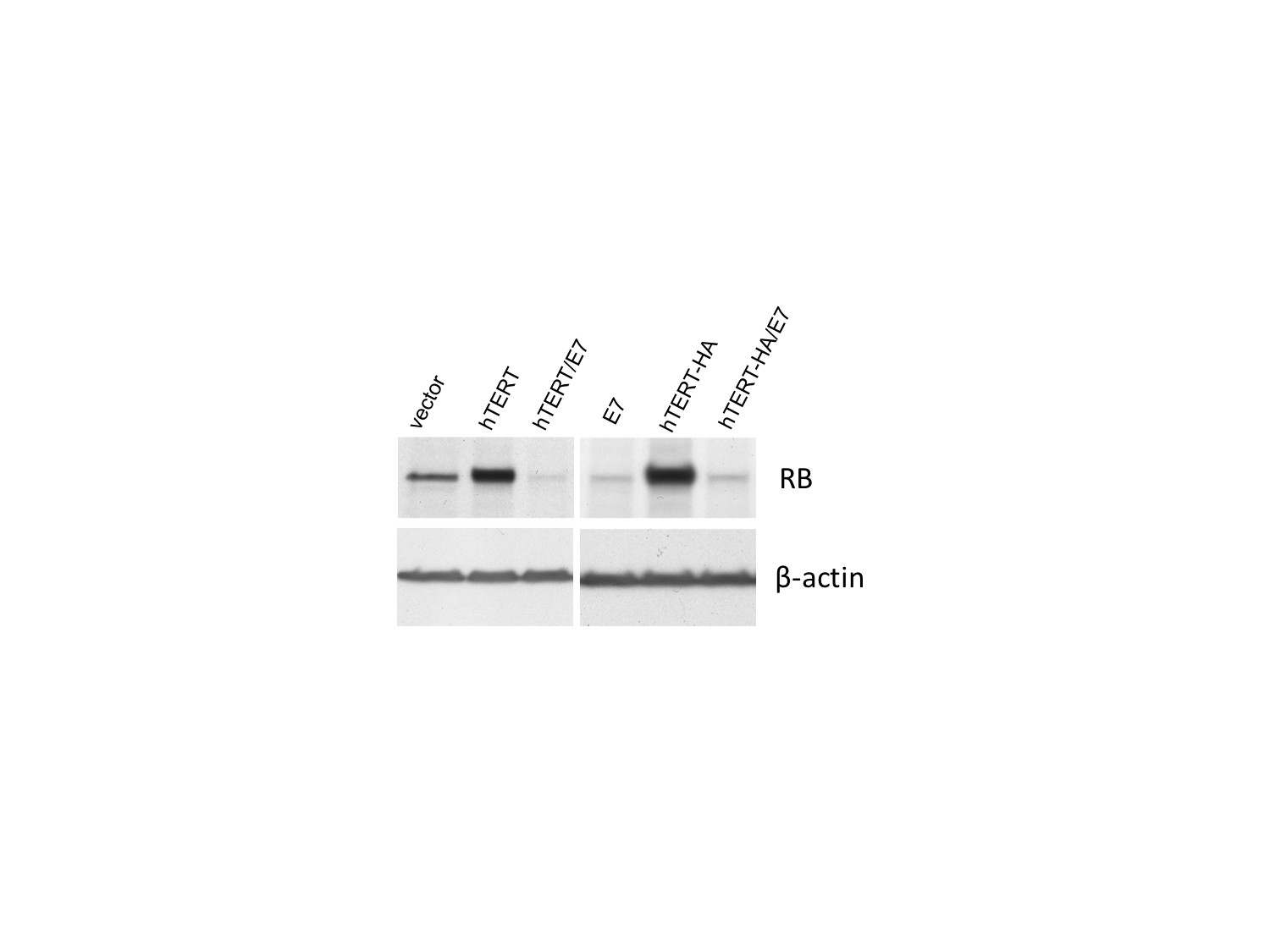

Supplement: Figure S1 — pRb levels validate the expression of a functional E7 protein. Cell lysates were extracted with 2x SDS buffer and subjected to SDS-PAGE gel and blotted with anti-Rb antibody. β-actin was used as internal control. pRb level decrease in E7, hTERT/E7 and hTERT-HA/E7 expressing cells. (TIF) [file ppat.1003284.s003.tif]

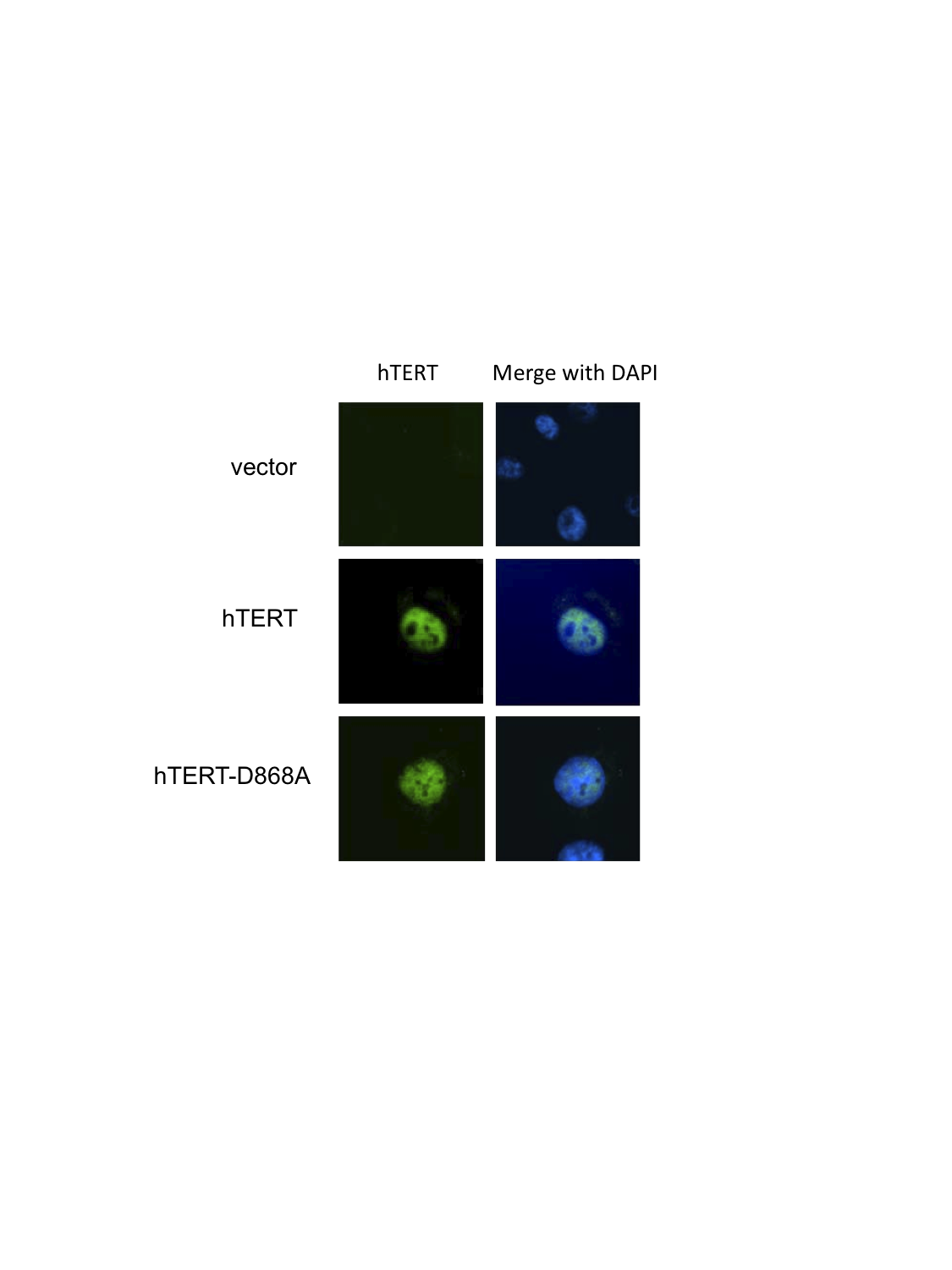

Supplement: Figure S2 — Wild-type and hTERT mutants localize to the nucleus. The hTERT-D868A mutant expresses and localizes in the nucleus similar to wild-type hTERT. (TIF) [file ppat.1003284.s004.tif]

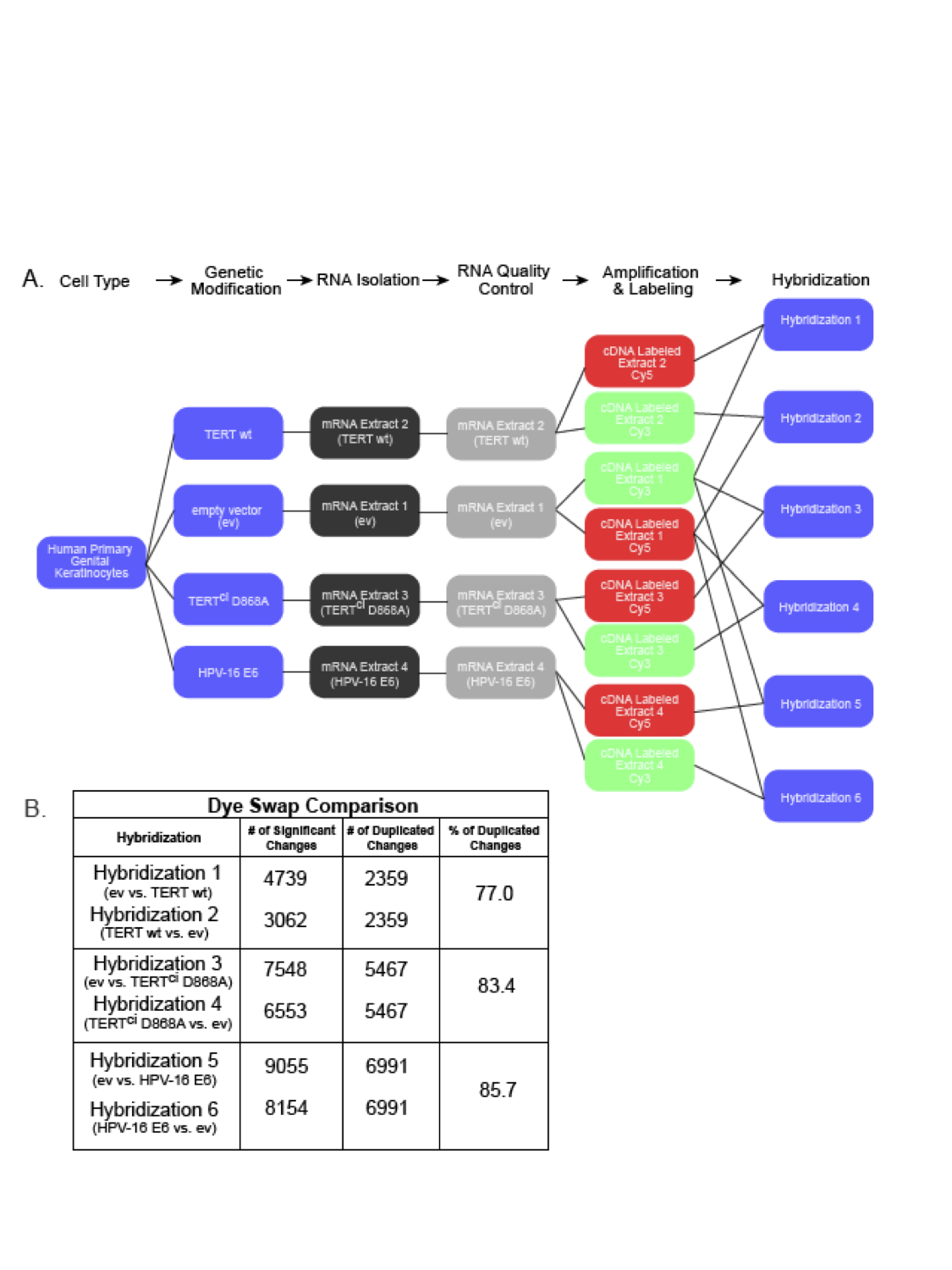

Supplement: Figure S3 — Scheme of array-based whole genome expression analysis. (A) We stably expressed E6, hTERT wt or a catalytically inactive mutant hTERT (D868A) in primary HFKs. Cells were lysed using the TRIzol reagent and RNA isolated following the manufacturer's protocol (Invitrogen) from samples 12–14 days post-infection. RNA quality was assessed by bioanalyzer. RNA was reverse transcribed and labeled with fluorescent dyes (Cy3 or Cy5) and submitted for array analysis using the Agilent 4 x 44K format. (B) Dye swap comparisons were made and directionally consistent changes were identified. The high percentage of consistency in expression changes are shown in the chart and support the integrity of the data. (TIF) [file ppat.1003284.s005.tif]

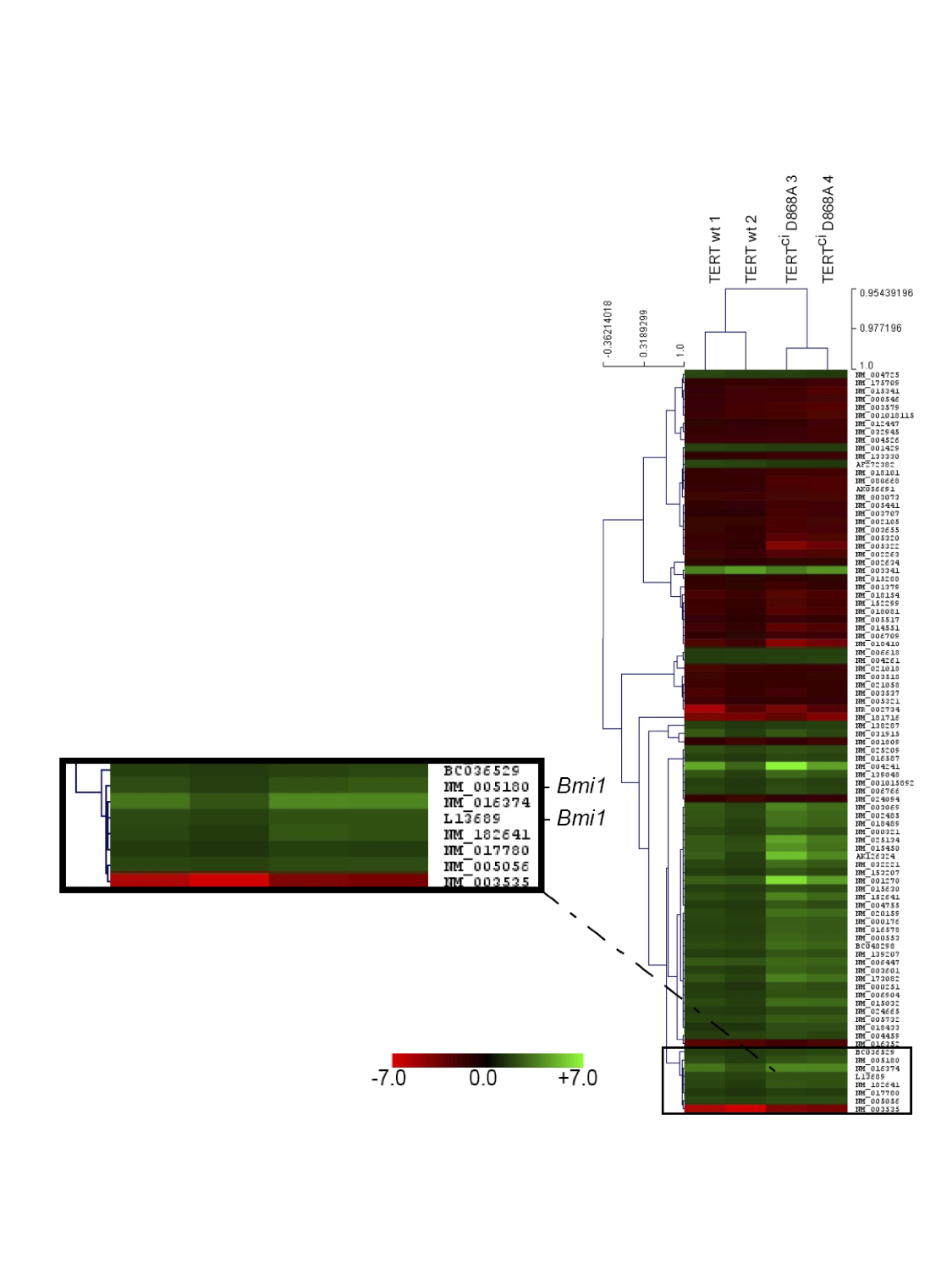

Supplement: Figure S4 — Visual representation of chromatin remodeling genes altered by hTERT. After identification of the chromatin cluster through functional annotation analysis, a heat map visualizing this cluster of probes was constructed. The four TERT arrays (two arrays for hTERT wt, two arrays for hTERT-D868A, duplicate samples with dye swap). Red represents a decrease in fold change vs empty vector (ev) while green represents an increase in fold change vs ev, and black represents no change. Intensity of color correlates to intensity of fold change, as described by the scale bar. Of the ninety one probes, Bmi1 is highlighted. (TIF) [file ppat.1003284.s006.tif]

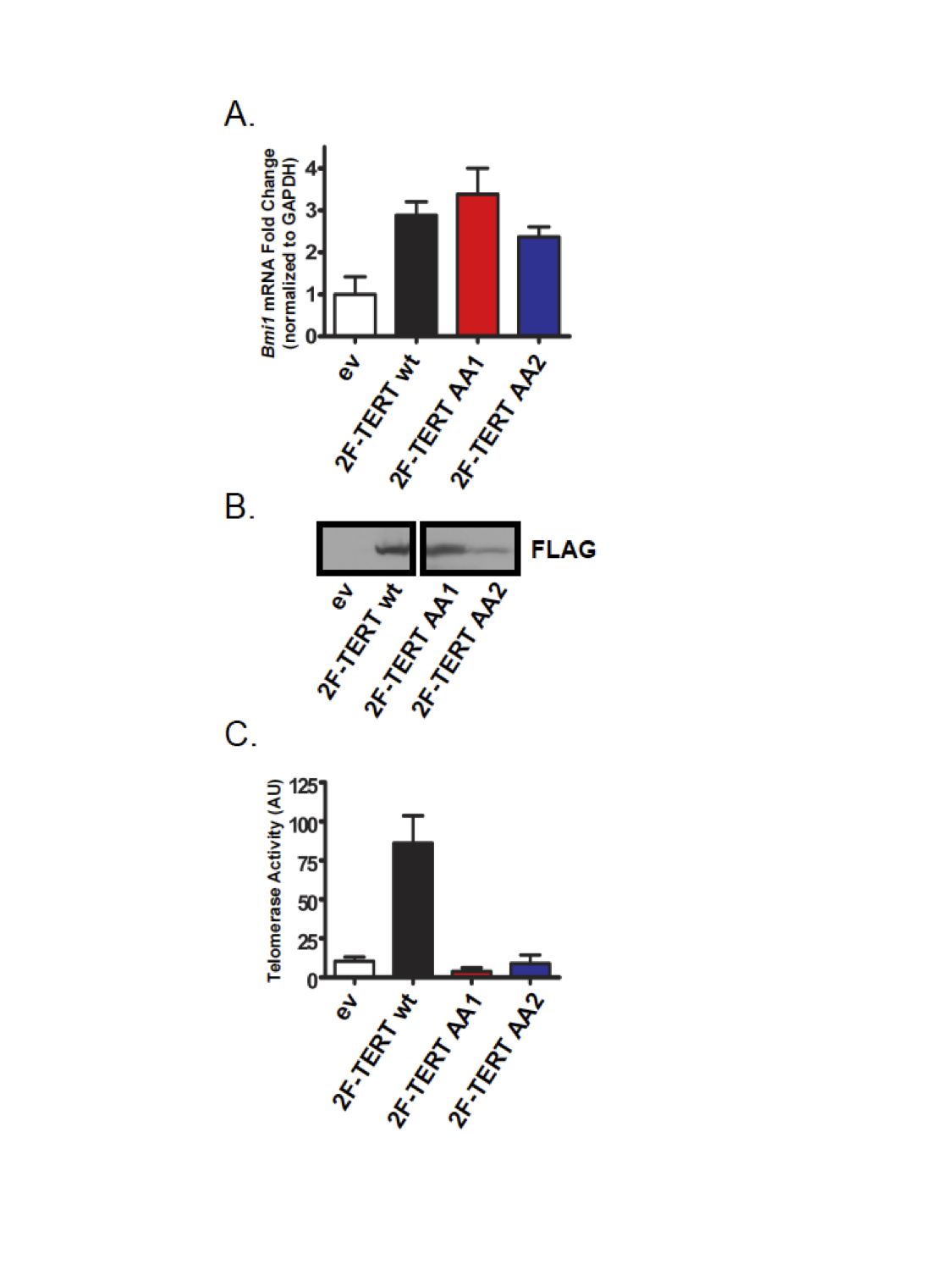

Supplement: Figure S5 — Bmi1 mRNA and Bmi1 protein increase with the expression of inactive hTERT mutants. To further validate Bmi1 increases seen by hTERT wt and hTERT-D868A expressing cells, additional hTERT mutants were also tested to determine if their expression increased Bmi1 mRNA levels. (A) Quantitative RT-PCR was performed with gene-specific primers for Bmi1, normalized to GAPDH. n = 3. Bars represent mean ± SD. 2F represents a double FLAG epitope tag on the N-terminus of the hTERT protein. Mutant AA1 had two leucine to alanine point mutations made at residues 837 and 840 (L837A, L840A) while AA2 had two leucine to alanine point mutations made at residues 863 and 866 (L863A, L866A). (B) Western blot confirmation of hTERT expression using a FLAG antibody for detection (1∶1000, Sigma). Samples were run on the same 4–20% gradient SDS-PAGE but separated by several lanes. Image cropped accordingly. (C) Quantitative TRAP confirms positive TRAP activity in hTERT wt and suggests mutants AA1 and AA2 are TRAP negative, compared to empty vector alone. (TIF) [file ppat.1003284.s007.tif]

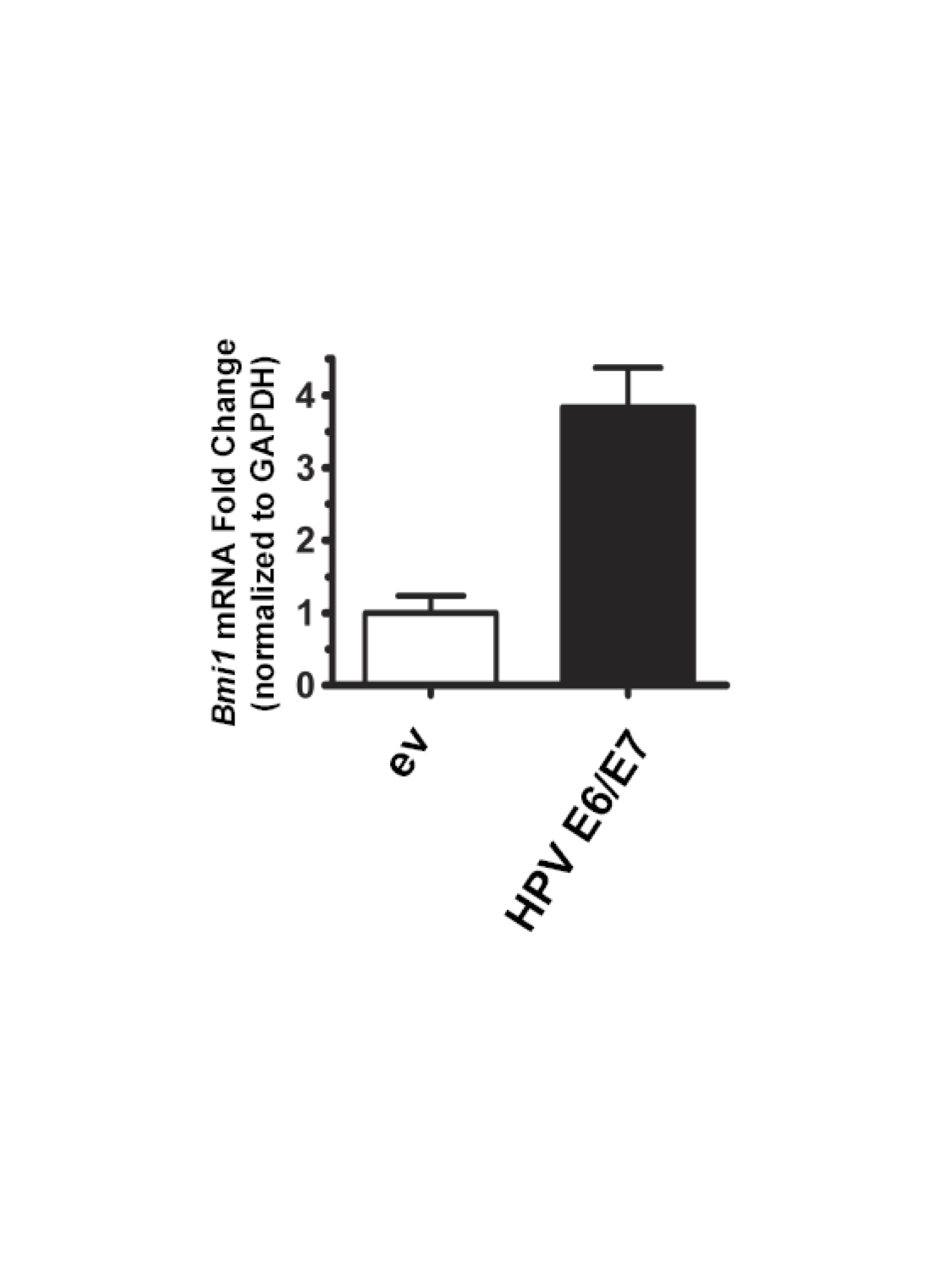

Supplement: Figure S6 — Bmi1 mRNA is increased in E6/E7 HFKs compared to empty vector alone. Quantitative RT-PCR was performed on empty vector and E6/E7 HFKs with gene-specific primers for Bmi1, normalized to GAPDH. n = 3. Bars represent mean ± SD. (TIF) [file ppat.1003284.s008.tif]
