# Supplementary material for: HPV16 E7 Protein and hTERT Proteins Defective for Telomere Maintenance Cooperate to Immortalize Human Keratinocytes
Source: PLoS Pathog. 2013 Apr 4;9(4):e1003284. doi: 10.1371/journal.ppat.1003284 (PMC3617164; doi:10.1371/journal.ppat.1003284)
Supplement: Table S1 — Properties of cells immortalized by hTERT, E6, and E7. (PDF) [file ppat.1003284.s009.pdf]

	hTERT Expression	Telomerase activity	Telomere Elongation	P53	RB/p16	Immortal
**HFK**						
E6/E7	+	+	-	-	-	+
E6	+	+	-	-	+	-
E7	-	-	-	+	-	-
hTERT	+	+	+	+	+	-
Myc	+	+	-	+	+	-
Myc/E7	+	+	-	+	-	+
hTERT/E7	+	+	+	+	-	+
hTERT-HA/E7	+	+	-	+	-	+
hTERTD868A/E7	+	-	-	+	-	+

+ functional or protein epxressed; - inactive or protein degraded
